# Solution‐Phase Energy Decomposition Analysis (SP‐EDA): Theory and Applications

**DOI:** 10.1002/jcc.70451

**Published:** 2026-07-17

**Authors:** Gábor Paragi, Célia Fonseca Guerra, F. Matthias Bickelhaupt

**Affiliations:** ^1^ Institute of Physics, University of Pécs Pécs Hungary; ^2^ Department of Theoretical Physics University of Szeged Szeged Hungary; ^3^ Institute of Medicinal Chemistry, University of Szeged Szeged Hungary; ^4^ Department of Chemistry and Pharmaceutical Sciences, AIMMS Vrije Universiteit Amsterdam Amsterdam the Netherlands; ^5^ Institute of Molecules and Materials, Radboud University Nijmegen the Netherlands; ^6^ Department of Chemical Sciences University of Johannesburg Johannesburg South Africa

**Keywords:** bond theory, density functional calculations, energy decomposition analysis, MO theory, solution phase

## Abstract

We present an extension of our canonical energy decomposition analysis (EDA) from the gas phase to a Solution‐Phase approach (SP‐EDA), that is, to interactions between molecular fragments in the condensed phase, simulated using the Conductor‐like Screening Model (COSMO). To this end, we have introduced different solvation states that serve to simulate particular physical phenomena that can be associated with the step from separate solvated fragments to the solvated complex of these fragments. In addition to the well‐known deformation strain of the fragments, induced by complex formation, one can distinguish the desolvation of the contact points via which the fragments bind, the response of the solvent to the deformation of the fragments as well as the response of the solvent to the formation of the complex, and finally the complex formation between the partially desolvated fragments. We provide definitions of how to model these phenomena using a continuum model, such as COSMO. Finally, we analyze, for representative model systems, how the bonding and, in particular, the EDA terms of electrostatic attraction, Pauli repulsion, and stabilizing orbital interactions behave across the above steps associated with interactions in solution. An important practical conclusion is that neglecting the effect of the partial desolvation at the binding sites has, in most cases, only minor consequences for the values and trends in the EDA terms. This finding can be used to simplify investigations using SP‐EDA.

## Graphical abstract

*Solution!* The SP‐EDA method has been developed to extend the energy decomposition analysis (EDA) of bond energies from gas‐phase to solution‐phase (SP) conditions. We evaluate and interpret various conceivable choices for bringing the solvation state stepwise from the initial fragment state (S0) to the final bound‐molecule state (S4) and assess how these choices affect the values of the bond‐energy components ∆*V*_elstat_, ∆*E*_Pauli_, and ∆*E*_oi_.

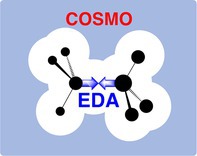

## 1 Introduction

A minute understanding of the chemical bond is a prerequisite for the ability to rationally manipulate molecular architecture at will. This requires a physically sound, predictive model of the chemical bond; a model that constitutes a causal, mechanistic relationship between, on the one hand, electronic and molecular structure and, on the other hand, stability and other properties. But it also requires that the various features in a bonding mechanism can be quantified, especially if one is interested in subtle effects.

One of the most successful models in the theory of chemical bonding and reactivity has been molecular orbital (MO) theory [1, 2]. Kohn‐Sham density functional theory (DFT) constitutes a quantitative, canonical MO theory which is in principle even exact although, in practice, it is as accurate as the density functional approximation that one employs [3]. Our canonical energy decomposition analysis (EDA), which facilitates the aforementioned quantification of individual terms in the bonding mechanism, was first introduced in DFT almost 50 years ago by Ziegler and Rauk [4, 5]. Since, it has served the analysis of chemical bonds between closed‐shell molecular fragments, in the gas phase. In 1992, the EDA method was augmented with the capability to handle open‐shell molecular fragments [6] and, thus, for example, electron‐pair bonding.

However, the EDA method has been limited so far to chemical bonds in the gas phase. This is a severe limitation, given that many, if not most, chemical processes occur in solution. Consequently, the primary aim of the present work is to introduce a newly developed solution‐phase energy decomposition analysis (SP‐EDA) which extends the scope of our analysis scheme to condensed‐phase chemistry.

In computational simulations, solvent effects can be taken into account explicitly or implicitly. The explicit solvent approach is more realistic, but it suffers from several problems. For example, in quantum chemical calculations usually only a few molecules represent the solvent, although solvent molecules, especially polar ones, behave drastically differently, when they occur in large numbers as in a real solution. The complexity not only derives from the large number of solvent molecules but especially also from the fact that solvent structure is not static but fluctuates dynamically [7].

Implicit solvation models, by their nature, can handle this situation efficiently by simulating the average effect of a solvent medium that interacts noncovalently with the solute. Thus, in the Polarizable Continuum Models (PCMs) [8], explicit solvents are substituted by a polarizable continuum and the investigated complex is placed in a properly defined cavity within the continuum. The cavity surface carries the mirror charge distribution, which is induced by the electrostatic potential of the solute. This mirror charge distribution can be conceived as a representation or simulation of solvent structure, that is, the average presence and orientation of solvent molecules. Thus, interaction between solvated fragments can be examined by a properly selected EDA method.

Herein, we extend our canonical EDA method from interacting systems in vacuum to interacting systems in the condensed phase simulated by the Conductor‐like Screening Model (COSMO) [9, 10]. The COSMO scheme, like other PCMs, involves two principal constituents: (i) the definition of a solvent cavity in which the solute molecule is placed; and (ii) the determination of the induced point charges at the cavity surface (COSMO charges), which simulates the response of the solvent to the solute with which it interacts. We do pay much attention to the various choices that must be considered in the SP‐EDA as two fragments form a complex: (i) via which steps do the original solute‐fragment cavities transform their shape to that of the final cavity of the combined solute complex; and (ii) how does one have to treat the change in solvent state, upon complex formation between the solute fragments, as reflected by the COSMO charges on the cavity surfaces. All these aspects can and do affect the individual SP‐EDA terms. For example, we can have different values for electrostatic attraction, Pauli repulsion, or orbital interaction for one and the same complex formation in solution, depending on which solvation state was selected for the interacting fragments. Of course, this may affect the theoretical interpretation of the bonding mechanism. Thus, we will examine how the interpretation of solution‐phase bond formation depends on the various approaches to cavitation and changes in solvent state, using a number of different test sets of model systems.

We would like to note that although other EDA schemes have been introduced for PCMs, they are theoretically distinct decomposition techniques and therefore do not apply directly to our canonical EDA method. Other approaches include the vacuum Kitaura‐Morokuma EDA which was extended to implicit‐solvation situations by Cammi et al. [11]. Related approaches were later suggested by Contador et al. and Góra et al. [12, 13] Solution‐phase extensions have also been developed for pair‐interaction EDA within the FMO framework [14, 15, 16, 17], for LMO‐EDA [18] (and its Generalized Kohn‐Sham variant [19]), and for ALMO‐EDA in combination with PCM [20]. In addition, the fragment‐localized molecular‐orbital (FLMO) version of the Kohn‐Sham scheme (KS‐FLMO), introduced by Giovannini, provided the basis for the KS fragment EDA (KS‐FEDA), formulated both in vacuum and in solution [21].

Importantly, our SP‐EDA scheme is not only an extension of yet another EDA variant. Beyond the more technical aspect of enabling our canonical EDA to handle solution‐phase situations, and in contrast to previous studies, our main purpose is to investigate how the various steps in solvation states and the choices one can make affect the SP‐EDA terms. We analyze and discuss how the steps between solvation states should be interpreted, which choices are physically meaningful, and which are not. This has not been systematically examined before in the literature.

In the following, we first introduce the basics of the SP‐EDA method, together with the computational details. Thereafter, the SP‐EDA formalism will be applied to three types of situations. One of them is based on the well‐known water dimer, as it can cover a wide range of electrostatic cases, like the interaction between neutral fragments, H_2_O•••H_2_O, or a charged and a neutral one, that is, H_3_O^+^•••H_2_O and HO^−^•••H_2_O. The second test case is about oppositely charged ionic fragments using the Na^+^•••OH^−^ complex as an archetypal example. The third test case is the bonding mechanism in the guanine tetrad G_4_, which exhibits significant changes when going from the gas phase to the solution phase [22].

## 2 Solution‐Phase Energy Decomposition Analysis (SP‐EDA)

### 2.1 Activation Strain Model

The activation strain model (ASM) is a fragment‐based approach in which the change in the energy associated with a chemical reaction, that is, the potential energy surface along the reaction coordinate, is described with respect to, and understood in terms of, the characteristics of the original reactants [23, 24, 25, 26]. Here, a chemical reaction can refer to a simple bond formation that occurs in the absence of a transition state, or to a more complex chemical process involving such transition states and intermediates. In this model, the potential energy surface Δ*E*(ζ) is decomposed into two contributions along the reaction coordinate ζ: the reaction strain ∆*E*_strain_(ζ), which is associated with the structural deformation that the reactants undergo during the reaction, plus the interaction ∆*E*_int_(ζ) between these increasingly distorted reactants. Herein, we will focus solely on bond formation in equilibrium structures (ζ = ζ _equilibrium_) and, for clarity, we will drop therefore the reaction coordinate from our equations:

(1) $$\Delta E\;=\;\Delta E_{\text{strain}}+\Delta E_{\mathrm{int}}$$

In this equation, the total strain energy, ∆*E*_strain_, is the penalty that needs to be paid in order to deform the reactants (or molecular fragments), say, A + B, from their equilibrium structure to the geometry they adopt in the final molecule or complex that is formed in the bond formation reaction. The interaction energy accounts for the interactions that occur between these two deformed fragments in the final molecule. This interaction energy can be further analyzed as described in the following.

### 2.2 Energy Decomposition Analysis (EDA) in Vacuum

The interaction energy ∆*E*_int_ between the deformed fragments in vacuum can be further analyzed in terms of quantitative Kohn‐Sham molecular orbital theory (KS‐MO) together with a canonical energy decomposition analysis (EDA) [3]. In the original EDA method, developed for bond formation in the gas phase, this interaction and all terms into which it decomposes, occur for fragments and a complex in vacuum and thus in the absence of any solvent effects, such as, cavitation and solute–solvent interactions (this changes if bond formation happens in solution, *vide infra*). Thus, in the EDA scheme, the interaction energy ∆*E*_int_ is decomposed into the following four, physically meaningful energy terms:

(2) $$\Delta E_{\mathrm{int}}\;=\Delta V_{\text{elstat}}+\Delta E_{\text{Pauli}}+\Delta E_{\mathrm{oi}}+\Delta E_{\text{disp}}$$

Herein, ∆*V*_elstat_ is the classical electrostatic interaction between the unperturbed charge distributions of the deformed fragments. This term is the change in energy associated with going from the separate, deformed fragments with wavefunctions Ψ^A^ and Ψ^B^, the corresponding charge densities ρ^A^ and ρ^B^ and total electronic fragment energies *E* ^A^ and *E* ^B^ to the promolecule in which these fragments with their fragment wavefunctions and densities have adopted their position in the overall molecule. This promolecule is thus characterized by the Hartree wavefunction, that is, the product of the unperturbed reactant wavefunctions Ψ^H^ = Ψ^A^ Ψ^B^, the corresponding sum density ρ^A + B^ = ρ^A^ + ρ^B^, and the promolecule energy *E* ^A + B^. The classical electrostatic interaction ∆*V*_elstat_ = *E* ^A + B^—*E* ^A^—*E* ^B^, is given by the following expression:
(3)

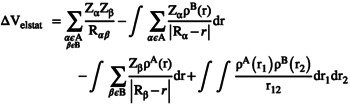

In Equation (3), the second and third terms represent the stabilizing interaction between the positive potential of the nuclei of one fragment with the electrons of the other fragment, while the first and fourth terms are the repulsive nucleus–nucleus and electron–electron interactions, respectively. When the two fragments are far apart, thus when ρ^A^ and ρ^B^ do not overlap, the resulting electrostatic interaction is, in the case of neutral reactants, zero. As soon as fragments A and B start to approach each other, and ρ^A^ and ρ^B^ begin to overlap, the electrostatic interaction between the unmodified charge distributions becomes increasingly more stabilizing until the point at which the repulsion between the nuclei becomes dominant [3, 27].

The Pauli repulsion ∆*E*_Pauli_ is calculated as the energy change of going from the Hartree wavefunction Ψ^H^, obtained in the first step, to the wavefunction Ψ^0^ = *N*Â{Ψ^H^}, which results from proper antisymmetrizing (operator Â) and renormalizing (constant *N*) the Hartree wavefunction. This ensures that Pauli's principle for fermionic wavefunctions is correctly satisfied for the overall system. Pauli repulsion arises between occupied spin‐orbitals of one fragment having overlap with occupied spin‐orbitals of the same spin on the other fragment. This mechanism is the origin of steric effects, that is, the physical phenomenon that matter occupies space. In practice, one can encounter this, for example, as the two‐center four‐electron destabilizing interactions between filled orbitals of the two reactants.

The orbital interaction energy ∆*E*_oi_ is computed in a final step by allowing the wavefunction Ψ^0^, with corresponding electronic density ρ^0^, to relax, through occupied–virtual mixing (e.g., HOMO–LUMO interactions between reactants and polarization within reactants), to the final wavefunction Ψ^AB^ and associated electronic density ρ^AB^ of the AB complex. If the two interacting fragments are open‐shell systems, the orbital interactions can also involve the formation of, e.g., an electron‐pair bonding configuration. The orbital interaction energy, ∆*E*_oi_, can be further decomposed into the contributions from each irreducible representation (irrep) Γ of the point group to which AB belongs:

(4) $$\text{∆}E_{\mathrm{oi}}=\Sigma_{\Gamma }\;\text{∆}{E^{\Gamma }}_{\mathrm{oi}}$$

The last term in Equation (2) is the energy change in the dispersion term due to the formation of the complex (see Computational Details).

### 2.3 SP‐EDA: Solvation States

Next, we extend the original EDA formalism to account for a system in which reactants in solution form a complex in solution. In this approach, the solvent environment is represented implicitly using the continuum model employed in the COSMO method. The presence of an implicit solvent introduces additional complexity, as not only the geometries of the molecular fragments undergo modifications during complex formation, but also the surrounding cavity and surface charge distributions change accordingly.

To address these solvent‐induced effects, we introduce new intermediate states within the EDA framework that reflect the evolving implicit solvent environment. This extension is conceptually analogous to the original EDA methodology, where complex formation proceeds via an intermediate state involving distorted fragments. The incorporation of additional intermediate states allows for the separation of pure solvent effects from changes associated with the redistribution of electronic density. Since the nature of primary and secondary bond formation is fundamentally governed by the electronic interactions between fragments, these newly introduced states make the various physical phenomena that occur upon complex formation in solution more explicit and allow for quantification. This also facilitates a chemically intuitive interpretation of the interactions occurring in a solvated environment.

**FIGURE 1:**
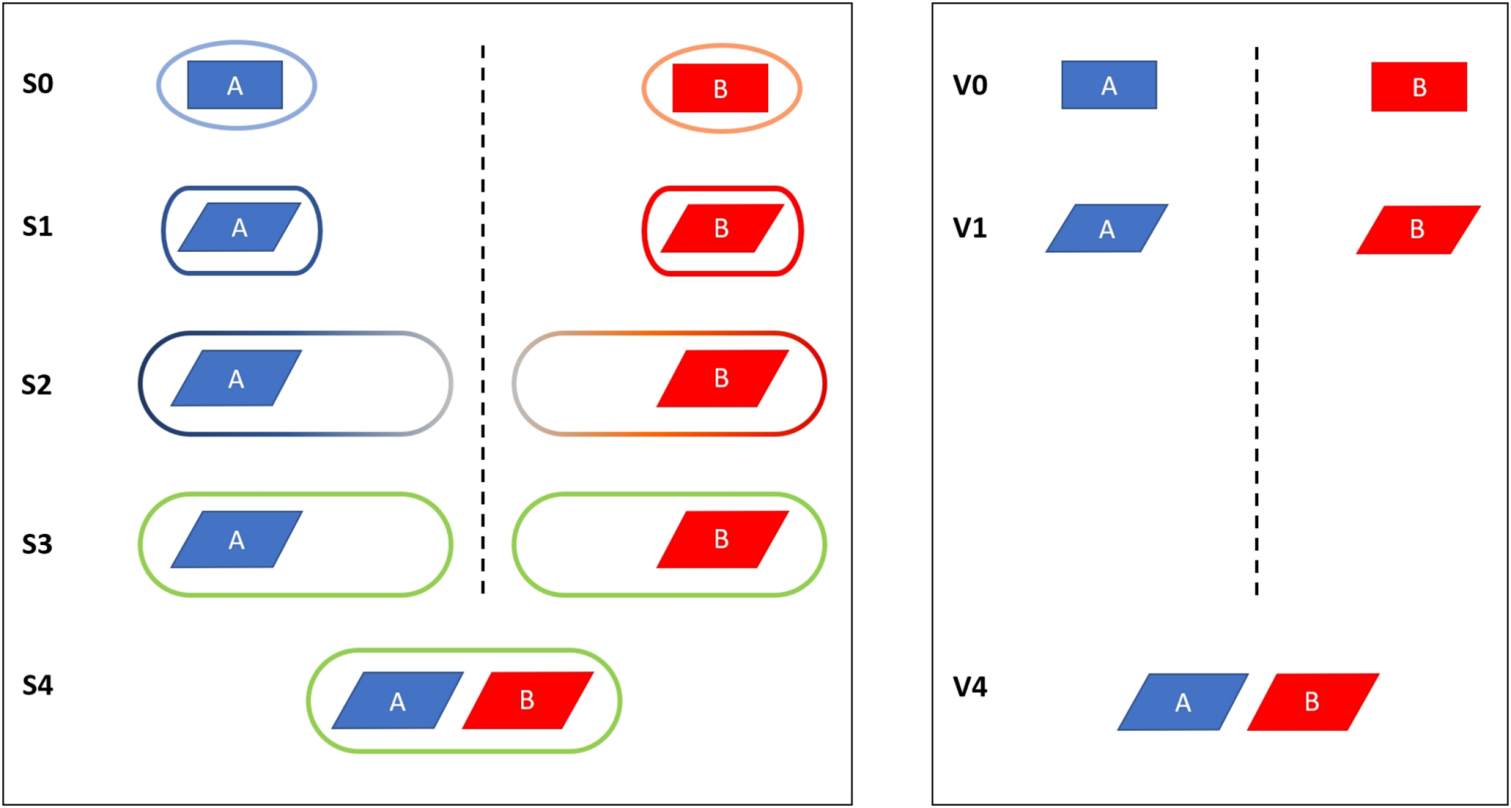
Schematic representation of the SP‐EDA solvation states S0–S4 for A + B forming AB in an implicit (COSMO) solvent (left) and the corresponding EDA vacuum states V0, V1, and V4 (right). The ovals around A, B, and AB in SP‐EDA represent solvent cavities, whereas colors designate the induced surface charges. S0 and V0: Separate A + B in their equilibrium geometry in solvent and in vacuum, respectively. S1 and V1: Separate A + B deformed to their geometry in the AB complex in solvent and in vacuum, respectively. S2: The deformed A + B in solvent as in S1, each in the AB cavity with fragment‐induced surface charges. S3: The same deformed A + B, each in the AB cavity with AB‐induced surface charges. S4 and V4: AB in the AB cavity with AB‐induced surface charges in solvent and AB in vacuum, respectively. There is no vacuum analogon for solvation states S2 and S3.

To illustrate this approach, Figure 1 shows a schematic representation of a two‐fragment complex in both implicit solvent and vacuum environments. The colored lines denote the boundaries of the solvation cavity and the COSMO surface charges surrounding the individual fragments and the complex as a whole. The proposed intermediate states, labeled S0 through S4, are defined in the following. S0 represents the individual fragments optimized in implicit solvent. S1 depicts the distorted fragments within their own implicit solvent environments, where the fragment geometries correspond to those found in the optimized solvated complex. S2 introduces the final solvation cavity size and shape of the solvated complexes, but used for both fragments.

While allowing the COSMO surface charges to remain unconstrained; thus, the surface charges adapt to the potential generated by the charge distributions of the respective fragments. S3 maintains the size and shape of the solvation cavities from S2, while the COSMO surface charges are adjusted to their final values as determined by the implicit solvent DFT calculation of the optimized solvated complex. Finally, S4 represents the fully optimized complex in its final COSMO solvation environment. In analogy to the S0—S4 states, we introduce the V0, V1, and V4 states for the original vacuum EDA. Note that in vacuum, there are no analogs of S2 and S3, that is, there are no V2 and V3 states.

The extended EDA approach provides a systematic means of decomposing and analyzing solvent effects on molecular interactions, enabling a more detailed understanding of the role of solvation in complex formation. In principle, any of the states S1–S3 may serve as a reference state for calculating interaction energies between fragments. However, we further investigate whether any of these states offer distinct advantages in terms of chemical interpretability.

### 2.4 SP‐EDA Energy Expressions

In the context of the SP‐EDA method, including an implicit solvent introduces additional terms to the conventional DFT total‐energy functional. This modification can be expressed as:

(5) $$E_{\text{COSMO}}\left[\rho \right]=E_{\mathrm{DFT}}\left[\rho \right]+E_{\text{solv}}\left[\rho \right]$$

Here, the energy E_DFT_ comprises the usual terms for a system in vacuum (i.e., the one‐electron kinetic energy, nuclear attraction, Coulomb energy, exchange‐correlation energy, and dispersion correction), but its value differs from the regular vacuum value because we substitute a different density into the functional, which density is optimized in the field of mirror charges induced on the cavity surface. The solvation energy *E*_solv_ accounts for the solvation contribution, which itself consists of two distinct components: (i) the cavitation term *E*_solv,cav_, which is proportional to the cavity surface area; and (ii) the electrostatic interaction term *E*_solv,elstat_[*ρ*], which arises as the interaction between the solute charge distribution and the COSMO mirror charges on the surface of the cavity (see Equation 6):

(6) $$\;E_{\text{solv}}\left[\rho \right]\;=E_{\text{solv},\mathrm{cav}}+E_{\text{solv},\text{elstat}}\left[\rho \right]$$

The total bonding energy Δ*E* of a complex is the energy difference between, on one hand, the total energy *E* ^S4^(AB) or *E* ^V4^(AB) of the final, bonded complex AB in its equilibrium geometry (state S4 or V4 in Figure 1) and, on the other hand, the sum of the total energies *E* ^S0^(A) + *E* ^S0^(B) [or *E* ^V0^(A) + *E* ^V0^(B)] of the isolated fragments A and B in their respective equilibrium geometries (state S0 or V0 in Figure 1); see Equation (7a). Both, in the vacuum and in the solution‐phase EDA, there is an intermediate state which corresponds to the phenomenon that the original reactants or fragments A and B undergo a geometrical deformation when they interact and form the complex AB. This geometrical deformation goes with a change in energy: the strain energy ∆*E*_strain_. Note that in the solution phase, this strain energy refers to a change from solvated fragments in their equilibrium geometry in solution to deformed fragments in solution and, therefore, already includes an effect of solvation. These solvation effects account for the fact that both, the cavity changes its shape and size (causing a change in cavitation energy: ∆*E*_solv,cav_) as well as the charge distribution of the solute and the mirror charges on the cavity surface change, due to the structural distortion (causing a change in the interaction between the solute charge distribution and the mirror charges: ∆*E*_solv,elstat_). The step from deformed fragments to the final molecular complex AB is then the interaction energy ∆*E*_int_. In the case of a solution‐phase process, this interaction energy, of course, also contains solvation effects (*vide infra*). These steps from S0 to S1 to S4 (or from V0 to V1 to V4 in vacuum) are summarized in Figure 1, and the corresponding energy differences are defined in Equations 7, which still apply to both a process in vacuum and a process in solution:

(7a) $$\text{∆}E=E^{\text{M4}}\left(\mathrm{AB}\right)–\left[E^{\text{M0}}\left(A\right)+E^{\text{M0}}\left(B\right)\right]$$

(7b) $$\begin{array}{c} =E^{\text{M4}}\left(\mathrm{AB}\right)–\left[E^{\text{M1}}\left(A\right)+E^{\text{M1}}\left(B\right)\right]+\left[E^{\text{M1}}\left(A\right)+E^{\text{M1}}\left(B\right)\right]\\ \;–\left[E^{\text{M0}}\left(A\right)+E^{\text{M0}}\left(B\right)\right] \end{array}$$

(7c) $$=\text{∆}E_{\mathrm{int}}+\text{∆}E_{\text{strain}}$$

where *M* = *S* or *V*, and with

(7d) $$\begin{array}{c} \text{∆}E_{\mathrm{int}}\equiv \Delta E_{\mathrm{int},M4-M1}=E^{\text{M4}}\left(\mathrm{AB}\right)–\left[E^{\text{M1}}\left(A\right)+E^{\text{M1}}\left(B\right)\right] \end{array}$$

(7e) $$\begin{array}{c} \text{∆}E_{\text{strain}}=\Delta E_{\text{strain},M1-M0}=\left[E^{\text{M1}}\left(A\right)+E^{\text{M1}}\left(B\right)\right]–\left[E^{\text{M0}}\left(A\right)+E^{\text{M0}}\left(B\right)\right] \end{array}$$

In the case of a bond formation in solution, two additional physical phenomena need to be considered that do not occur in vacuum: (i) change in cavitation; and (ii) partial desolvation. The change in cavitation refers to having different cavities in the solvent for the original fragments or reactants A and B, for the deformed fragments, and, finally, for the complex AB. This effect is associated with a change in energy, which is already accounted for in the cavitation term *E*_solv,cav_ (see Equation 6). The change in cavitation is also represented in Figure 1 by the change of the schematic cavities if one goes from state S0 to S1 to S4.

The phenomenon of partial desolvation refers to the desolvation of the contact points of the deformed fragments that takes place before these fragments can approach and bind, via the aforementioned contact points, to form the complex AB. This phenomenon can be modeled by placing each of the deformed fragments at the position they adopt in the final complex AB in the cavity that is of the size and shape of the final complex AB, but in the absence of the other fragment. In this situation, the solvent at the contact point of either the deformed fragment A or B is displaced. In our scheme, shown schematically in Figure 1, this situation is simulated by the intermediate state S2 in which each of the two deformed fragments is present, in the absence of the other fragment, in the overall cavity of AB. In state S2, the fragment experiences the mirror charges on the overall cavity induced by that fragment. This implies that the electronic structure of each fragment is influenced by the solvent by which it is surrounded, except for the region facing the empty space in the overall cavity that stands for the desolvated contact points. Proceeding from here, in state S2, the partially desolvated fragments interact and go to state S4, that is, they form the final complex AB in the overall cavity of AB with the mirror charges belonging to that final complex.

With the introduction of state S2, we can now decompose the interaction energy ∆*E*_int_ in solution into the two terms discussed above, as shown in Equations 8: (i) the partial‐desolvation energy $\Delta E_{\text{DESOLV},S2-S1}$ of the deformed fragments or reactants between state S1 and S2; and (ii) the interaction energy $\Delta E_{\mathrm{int},S4-S2}$ between these partially desolvated fragments in the final complex AB.

(8a) $$\begin{array}{c} \text{∆}E_{\mathrm{int}}\equiv \Delta E_{\mathrm{int},S4-S1}=E^{\text{S4}}\left(\mathrm{AB}\right)–\left[E^{\text{S1}}\left(A\right)+E^{\text{S1}}\left(B\right)\right] \end{array}$$

(8b) $$=E^{\text{S4}}\left(\mathrm{AB}\right)–\left[E^{\text{S2}}\left(A\right)+E^{\text{S2}}\left(B\right)\right]+\left[E^{\text{S2}}\left(A\right)+E^{\text{S2}}\left(B\right)\right]–\left[E^{\text{S1}}\left(A\right)+E^{\text{S1}}\left(B\right)\right]$$

(8c) $$=\Delta E_{\mathrm{int},S4-S2}+\Delta E_{\text{DESOLV},S2-S1}$$

with

(8d) $$\begin{array}{c} \Delta E_{\mathrm{int},S4-S2}=E^{S4}\left(\mathrm{AB}\right)–\left[E^{S2}\left(A\right)+E^{S2}\left(B\right)\right] \end{array}$$

(8e) $$\begin{array}{c} \Delta E_{\text{DESOLV},S2-S1}=\left[E^{S2}\left(A\right)+E^{S2}\left(B\right)\right]–\left[E^{S1}\left(A\right)+E^{S1}\left(B\right)\right] \end{array}$$

For completeness, we also introduce an alternative intermediate state S3, which has geometrically the same cavity, but the surface of this cavity exhibits the mirror charges induced by the final complex AB in that cavity. Thus, a single deformed fragment A or B of state S3 does not interact with its own mirror charge, which it would induce in the larger cavity, but with the mirror charge that the final complex induces in this cavity. The energy terms relating to a decomposition of the interaction energy ∆*E*_int_ using state S3 are shown in Equations 9. We will discuss the meaning of state S3 later on in the applications section. But already at this point, we anticipate that, despite some interesting considerations, state S3 is physically awkward and not helpful in interpreting bonding in the solution phase.

(9a) $$\text{∆}E_{\mathrm{int}}\equiv \text{∆}E_{\mathrm{int},S4-\text{S1}}=E^{\text{S4}}\left(\mathrm{AB}\right)–\left[E^{\text{S1}}\left(A\right)+E^{\text{S1}}\left(B\right)\right]$$

(9b) $$=E^{\text{S4}}\left(\mathrm{AB}\right)–\left[E^{\text{S3}}\left(A\right)+E^{\text{S3}}\left(B\right)\right]+\left[E^{\text{S3}}\left(A\right)+E^{\text{S3}}\left(B\right)\right]–\left[E^{\text{S1}}\left(A\right)+E^{\text{S1}}\left(B\right)\right]$$

(9c) $$=\Delta E_{\mathrm{int},S4-S3}+\Delta E_{\text{DESOLV},S3-S1}$$

with

(9d) $$\begin{array}{c} \Delta E_{\mathrm{int},S4-S3}=E^{S4}\left(\mathrm{AB}\right)–\left[E^{S3}\left(A\right)+E^{S3}\left(B\right)\right] \end{array}$$

(9e) $$\begin{array}{c} \Delta E_{\text{DESOLV},S3-S1}=\left[E^{S3}\left(A\right)+E^{S3}\left(B\right)\right]–\left[E^{S1}\left(A\right)+E^{S1}\left(B\right)\right] \end{array}$$

In vacuum, the interaction ∆*E*_int_ ≡ ∆*E*_int,V4‐V1_ is always between the deformed fragments of state V1 and the final complex state V4, and ∆*E*_int_ can be straightforwardly decomposed into chemically meaningful terms (Δ*V*_elstat_, Δ*E*_Pauli_, Δ*E*_oi_ and Δ*E*_disp_) using the energy decomposition analysis (EDA) described above. However, when evaluating $\Delta E_{\mathrm{int},S4-\mathrm{SX}}$ (where X = 1, 2, 3) in the presence of a solvent, the values of these EDA terms differ depending on the selected solvation state. This variation arises because EDA terms are derived from fragment densities, which have been influenced by solvation effects.

Thus, in the case of SP‐EDA calculations, that is, EDA calculations of interacting systems in solution, the interaction energy also includes contributions from solvation. However, this situation can be readily addressed, given that solvation energy is an additive term in the total energy expression, as previously pointed out (see Equation 5). Thus, we can write the new interaction energy expressions in a general way, including the partially desolvated states as well, as follows:

(10a) $$\begin{array}{c} \Delta E_{\mathrm{int},S4-\mathrm{SX}}=E^{S4}\left(\mathrm{AB}\right)–\left[E^{\mathrm{SX}}\left(A\right)+E^{\mathrm{SX}}\left(B\right)\right] \end{array}$$

(10b) $$\begin{array}{c} \;={E^{\text{S4}}}_{\mathrm{DFT}}\left(\mathrm{AB}\right)–\left[{E^{\mathrm{SX}}}_{\mathrm{DFT}}\left(A\right)+{E^{\mathrm{SX}}}_{\mathrm{DFT}}\left(B\right)\right]\\ \;+{E^{\text{S4}}}_{\text{solv}}\left(\mathrm{AB}\right)–\left[{E^{\mathrm{SX}}}_{\text{solv}}\left(A\right)+{E^{\mathrm{SX}}}_{\text{solv}}\left(B\right)\right] \end{array}$$

(10c) $$=\Delta E_{\mathrm{DFT},S4-\mathrm{SX}}+\Delta E_{\text{solv},S4-\mathrm{SX}}$$

where X = 1, 2 or 3, and with

(10d) $$\Delta E_{\mathrm{DFT},S4-\mathrm{SX}}=\Delta V_{\text{elstat},S4-\mathrm{SX}}+\Delta E_{\text{Pauli},S4-\mathrm{SX}}+\Delta E_{\mathrm{oi},S4-\mathrm{SX}}+\Delta E_{\text{disp}}$$

(10e) $$\begin{array}{c} \Delta E_{\text{solv},S4-\mathrm{SX}}={E^{S4}}_{\text{solv}}\left(\mathrm{AB}\right)–\left[{E^{\mathrm{SX}}}_{\text{solv}}\left(A\right)+{E^{\mathrm{SX}}}_{\text{solv}}\left(B\right)\right] \end{array}$$

**FIGURE 2:**
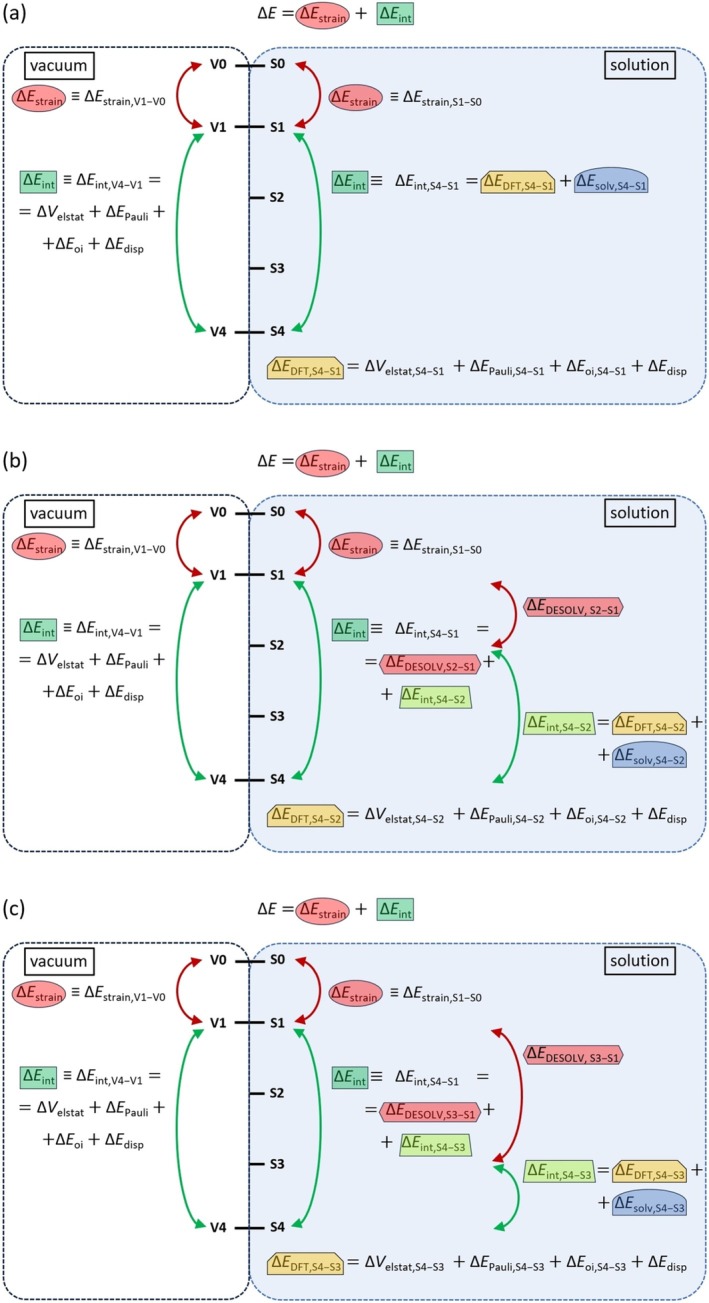
Graphical summary of the newly introduced SP‐EDA terms and the traditional vacuum decomposition. (a–c) illustrate the relationship between the original vacuum case and the different intermediate solution states S1, S2, and S3, respectively.

Thus, the total interaction energy $\Delta E_{\mathrm{int},S4-\mathrm{SX}}$ in solution is decomposed into the intrinsic interaction energy $\Delta E_{\mathrm{DFT},S4-\mathrm{SX}}$ between the solute fragments in the solute complex treated through DFT computations plus the differential solvation term $\Delta E_{\text{solv},S4-\mathrm{SX}}$ associated with the difference in solvation of the final state S4 and the initial state SX. The intrinsic interaction energy $\Delta E_{\mathrm{DFT},S4-\mathrm{SX}}$ can be decomposed into the regular EDA terms [4, 5] of the quantum‐chemically (DFT) described system in the solution‐phase situation, that is, $\Delta V_{\text{elstat},S4-\mathrm{SX}},\Delta E_{\text{Pauli},S4-\mathrm{SX}}$, and $\Delta E_{\mathrm{oi},S4-\mathrm{SX}}$, plus the dispersion correction ∆*E*_disp_ which is not affected by solution. We explore these terms and their interpretation using selected model systems in the following. In particular, we address which one of the intermediate states, S1, S2, or S3, is optimal for achieving a physically meaningful interpretation of the bonding mechanism in the solution phase. As mentioned before, state S3 is not among the recommended options. In Figure 2, we present a comprehensive graphical overview of all energy terms discussed so far. Figure 2a–c illustrate the relationship between the original vacuum case and the different intermediate solution states S1, S2, and S3, respectively.

## 3 Application 1: Complexes of Water With Hydroxide, Water, or Hydroxonium

Our first series of model systems for probing the interpretation of the three intermediate states S1, S2, and S3 in our SP‐EDA consists of the complexes of water with a hydroxide anion (HO^—^•••H_2_O), with another water (H_2_O•••H_2_O), and with a hydroxonium cation (H_3_O^+^•••H_2_O). This series has several assets. First, the interacting species are small and, therefore, have a relatively large surface‐to‐volume ratio, implying relatively strong exposure to the solvent medium and strong solvation effects. Secondly, the transition from a molecule–anion via neutral molecule–molecule to molecule cation complexes goes with substantial variation in the solvent cavities and mirror charges. This reflects prominently the differences in the interaction terms of such different complexes in solution. Interestingly, it also amplifies the different behavior in such situations of choosing different intermediate states and thus puts these choices to a stringent test.

The main effect of solvation is a weakening of the bond energies ∆*E*, especially for the ionic complexes: from −35.3 to −10.8 kcal/mol for HO^—^•••H_2_O, from −5.4 to −4.0 kcal/mol for H_2_O•••H_2_O, and from −37.1 kcal/mol to −15.8 kcal/mol for H_2_O^+^•••H_2_O (see Table 1). For the ionic complexes, the most important reason is the weakening of orbital interactions Δ*E*_oi,S4‐SX_ and, to a lesser extent, of electrostatic attraction Δ*V*_elstat,S4‐SX_, as solvation stabilizes the concentration of a net charge in the fragments. In the neutral complex, orbital interactions are slightly stabilized by solvation, which stabilizes the charge separation in the overall complex. In the neutral complex, the weakening stems entirely from a small but destabilizing differential solvation effect. Pauli repulsion terms are relatively constant if one goes from the gas phase to the solution phase. This can be ascribed to the relatively small change in shape of the occupied orbitals, which therefore generate similar closed‐shell overlap in either phase.

**TABLE 1:** EDA and SP‐EDA energy terms (in kcal/mol) for the HO^−^˙˙˙H_2_O, H_2_O˙˙˙H_2_O and H_3_O^+^˙˙˙H_2_O complexes with binding energies and O˙˙˙H bond distances (in Å).^a^

SP‐EDA (EDA)	HO ^−^ ˙˙˙H_2_O				H_2_O˙˙˙H_2_O				H_3_O^+^˙˙˙H_2_O			
	COSMO			Vac.	COSMO			Vac.	COSMO			Vac.
	S1	S2	S3	V1	S1	S2	S3	V1	S1	S2	S3	V1
Δ*V*_elstat,S4‐SX_ (in vac. Δ*V*_elstat_)	−53.2	−49.5	−46.9	−69.7	−12.1	−11.6	−11.5	−8.6	−48.2	−46.7	−39.6	−40.8
Δ*E*_Pauli,S4‐SX_ (in vac. Δ*E*_Pauli_)	47.4	47.2	50.3	85.1	11.7	11.8	11.7	8.7	58.9	61.0	57.4	54.7
Δ*E*_oi,S4‐SX_ (in vac. Δ*E*_oi_)	−34.0	−37.0	−42.8	−72.9	−4.5	−5.0	−4.3	−4.5	−62.1	−65.4	−68.5	−67.1
Δ*E* _disp_	−0.8	−0.8	−0.8	−0.6	−1.1	−1.1	−1.1	−1.1	−1.1	−1.1	−1.1	−1.1
Δ*E*_DFT,S4‐SX_ (or Δ*E*_int,V4‐V1_)^b^	−40.6	−40.1	−40.0	−58.2	−6.0	−5.8	−5.1	−5.5	−52.5	−52.2	−51.8	−54.3
Δ*E* _solv,S4‐SX_	24.1	18.1	−79.1	—	1.9	0.5	−17.2	—	19.7	13.9	−74.4	—
Δ*E*_int,S4‐SX_ (or Δ*E*_int,V4‐V1_)^c^	−16.5	−22.0	−119.1	−58.2	−4.1	−5.3	−22.3	−5.5	−32.8	−38.3	−126.2	−54.3
Δ*E*_DESOLV, SX‐S1_	0.0	5.6	102.6	—	0.0	1.1	18.1	—	0.0	5.6	93.5	—
Δ*E* _strain,M1‐M0_ (M = S or V)	5.6			22.8	0.1			0.1	16.9			17.3
Δ*E* ^d^	−10.8			−35.3	−4.0			−5.4	−15.8			−37.1
*O*˙˙˙*H dist*.	1.44			1.24	1.82			1.92	1.22			1.22

^a^ Computed at BLYP‐D/TZ2P. See Figure 2 for a graphical illustration of all energy terms.

^b^ Sum of the four EDA terms above this entry: c.f. (Equation 10d) (and Equation 2).

^c^ Sum of the two terms above this entry: *c.f*. (Equation (10a), (10b), (10c)) (and Equation 7d).

^d^ Bond energy (sum of the three terms above this entry: *c.f*. Equation 7a): the difference between states S4 and S0 (or V4 and V0 for vacuum).

**FIGURE 3:**
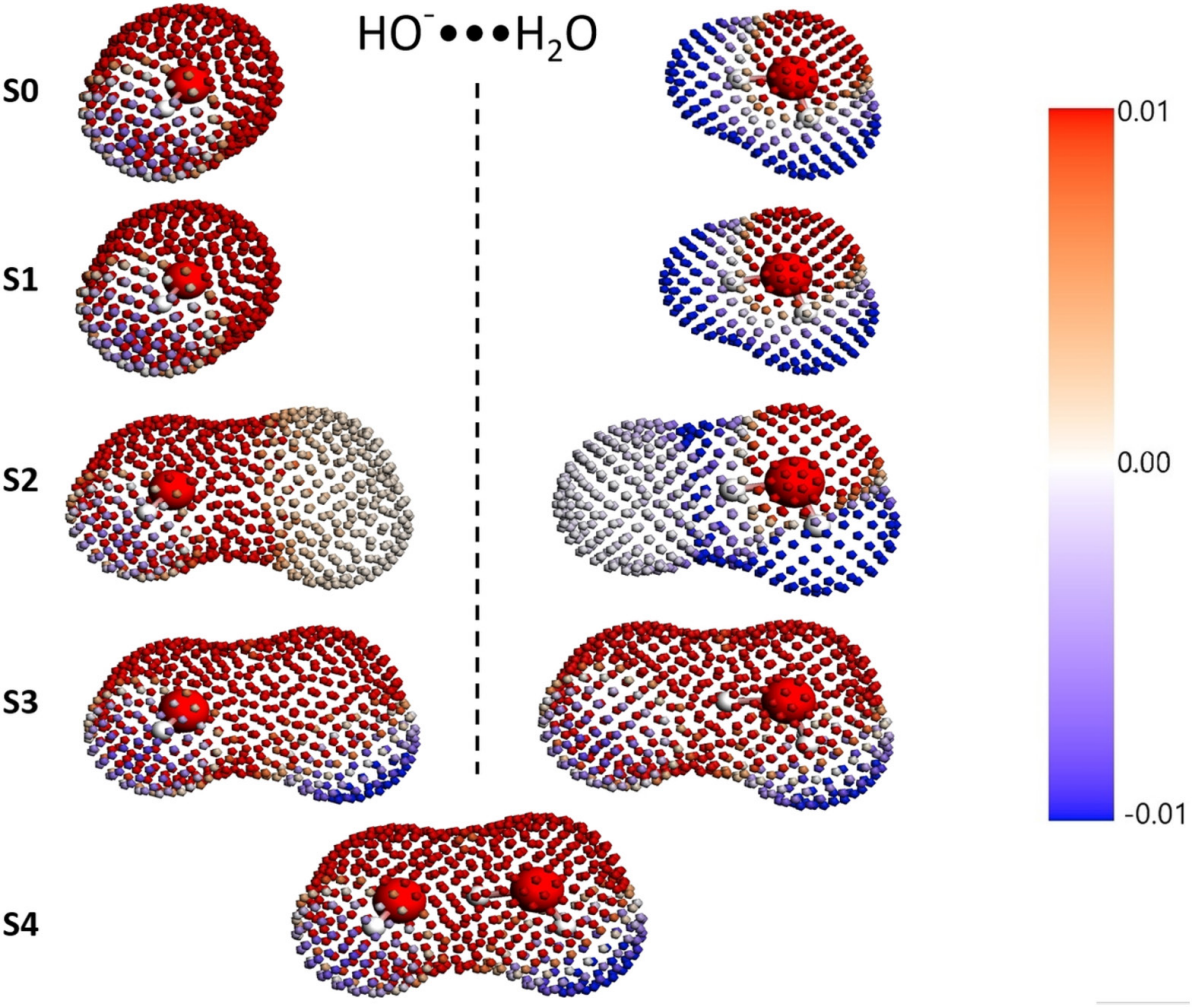
COSMO charge density of surface segments represented in the position of COSMO chargepoints for the HO^—^•••H_2_O complex and its fragments at different solvation states (S0–S4). Red, blue, and white indicate regions of positive, negative, and near‐neutral charge densities, respectively (see color scale in e/Å^2^ unit).

Figure 3 shows the cavity surfaces with COSMO densities, derived from mirror charges in states S0—S4 for the HO^—^•••H_2_O complex. Here, red points indicate the positive COSMO densities, blue color represents the negative ones, and gray/white points show the approximately neutral regions (see color scale in Figure 3). A comparison of the different solvation states reveals key trends. Minimal changes are observed concerning the solvent cavity between S0 and S1, though more considerable differences might emerge in systems with more distorted fragments. The transition from S1 to S2 results in cavity enlargement, affecting not only its size but also its charge distribution, as some surface segments become less charged when they are moved farther from the fragment in the enlarged cavity. This redistribution can influence fragment orbitals that are critical to intermolecular interactions, thereby altering the SCF procedure and the resulting electron density. The cavity enlargement is to be interpreted as a partial desolvation, that is, the exclusion of solvent from the inter‐fragment region during complex formation. Finally, significant differences arise between S2 and S3, where previously neutral regions become charged. This charge redistribution has a profound energetic impact, particularly for charged species. For example, in the HO^—^•••H_2_O system, the desolvation interaction energy $\Delta E_{\mathrm{int},S4-\mathrm{SX}}$ is found to be −22.0 and −119.1 kcal/mol for states SX = S2 and S3, respectively. Given that the vacuum interaction energy is −58.2 kcal/mol, the result for S3 appears unphysical, primarily due to an unrealistic solvation term (Table 1). The solvation term originates from the interaction of the solute charge distribution with COSMO charges, and the use of surface charges induced by the presence of a different solute system in the case of S3 (namely those belonging to the final HO^—^•••H_2_O complex), leads to artificial stabilization. Note that modifications in the implicit solvent affect not only the *E*_solv_ term in the total energy *E*_COSMO_ but also *E*_DFT_ (see Equation 5). The reason is that changes in the COSMO environment also alter the SCF densities in the quantum‐chemical DFT energy. This, in turn, influences SP‐EDA terms that derive from *E*_DFT_.

Comparison of interaction energies $\Delta E_{\mathrm{int},S4-\mathrm{SX}}$ within each of the three water complexes indicates that S1 and S2 behave similarly. This suggests that cavity enlargement, that is, the desolvation of the contact points on each fragment, does not drastically alter $\Delta E_{\mathrm{int},S4-\mathrm{SX}}$. However, employing both, the enlarged COSMO cavity and charge distribution in S3, significantly affects $\Delta E_{\text{solv},S4-S3}$_,_ and, consequently, $\Delta E_{\mathrm{int},S4-S3}$. Notably, the intrinsic interaction energy $\Delta E_{\mathrm{DFT},S4-S3}$ within the solute is less sensitive to COSMO cavity modifications than the differential solvation energy $\Delta E_{\text{solv},S4-S3}$. The additional partial desolvation energy required to go from S1 to higher solvation states $\Delta E_{\text{DESOLV},S3-S1}$ (see Table 1) is mainly a solvation effect and is notably larger for S3 than for S2. Given that interaction energies for S3 ($\Delta E_{\mathrm{int},S4-S3}$) exceed vacuum values (Δ*E*_int,V4‐V1_), S3 results are best conceived as originating from a COSMO environment that is incompatible with the fragments and therefore unrealistic or unphysical.

As mentioned earlier, the transition from S1 to S2 can be interpreted as the desolvation of the solute fragments at the positions through which they interact in the solute complex. Technically, this solvent displacement from the solute fragments also represents a small step toward vacuum conditions. Thus, the value of $\Delta E_{\mathrm{int},S4-S2}$ should fall between $\Delta E_{\mathrm{int},S4-S1}$ for the solution‐phase situation and Δ*E*_int,V4‐V1_ for the vacuum situation (see Table 1). This provides a clear physical interpretation for S2. State S3, on the other hand, has a less straightforward connection with physical phenomena. It represents a situation in which, in addition to desolvation of the solute fragments, the solvent is in the configuration (i.e., has the distribution of mirror charges) it adopts when it surrounds the final solute complex. This situation is electrostatically not optimal and corresponds to less favorable solvation of the solute fragments. The corresponding solution‐phase interaction energies $\Delta E_{\mathrm{int},S4-S3}$, therefore, have an unclear status and are, in general, significantly more stabilizing than the vacuum ∆*E*_int,V4‐V1_ (see Table 1).

We conclude that states S1 or S2 serve well for arriving at a physically well‐defined and interpretable solution‐phase EDA method, while S3 should be avoided. In line with this, we find that both choices, S1 and S2, yield similar pictures if one analyzes the bonding in the three water complexes. Thus, the bond energy ∆*E* increases from −4.0 kcal/mol for the neutral water dimer to −10.8 and −15.8 kcal/mol for the ionic complexes HO^—^•••H_2_O and H_3_O^+^•••H_2_O, respectively (see Table 1). This goes with a contraction of the O•••H bond distances from 1.82 Å for the water dimer to 1.44 and 1.22 Å for the two ionic complexes. The SP‐EDA analyses reveal that the strengthening of the bonds in the ionic complexes stems primarily from a stabilization of the orbital interactions, which are dominated by HOMO–LUMO interactions between an oxygen lone‐pair type orbital on one fragment (water or hydroxide) and an H–O σ* antibonding orbital on the other fragment (water or hydroxonium). Corresponding EDA terms (i.e., $\Delta V_{\text{elstat},S4-\mathrm{SX}},\Delta E_{\text{Pauli},S4-\mathrm{SX}}$, and $\Delta E_{\mathrm{oi},S4-\mathrm{SX}}$) agree well with each other (within 1–3 kcal/mol), whether one chooses states S1 or S2. Larger deviations can occur for state S3. Interestingly, the most significant deviations occurring in the case of state S3, namely, up to over 60 kcal/mol, stem from the differential solvation energy $\Delta E_{\text{solv},S4-\mathrm{SX}}$. This Reflects the unphysical status of a cavity in which a solute fragment interacts with a solvent that has already adopted its configuration to the complex, that is, in which the fragment experiences the solute complex's mirror charges.

## 4 Application 2: Sodium Hydroxide

As a second case study, we consider sodium hydroxide, NaOH. This system is particularly instructive because it represents the limiting case of a complex built from two oppositely charged fragments, Na^+^ and OH^−^. It therefore provides a stringent test of the solution‐phase EDA protocol introduced above. Whereas the water dimer systems discussed in the previous section allowed us to analyze neutral and singly charged hydrogen‐bonded complexes, NaOH enables us to examine how the method behaves when both fragments carry full formal charges of opposite sign.

We first optimized NaOH in the gas phase and performed a conventional EDA on this equilibrium structure. In aqueous solution, however, NaOH is not stable as a neutral contact ion pair but dissociates into Na^+^ and OH ^—^. To nevertheless enable a meaningful comparison between gas‐phase and solution‐phase energy terms, the solution‐phase geometry of the neutral NaOH complex was obtained by constrained optimization, keeping the Na–O distance fixed at the gas‐phase value of 2.016 Å. In this way, differences between the gas‐phase and solution‐phase EDA terms can be attributed to solvation effects rather than to changes in molecular geometry. On this constrained structure, we carried out single‐point EDA calculations using the different solvation states S1, S2, and S3 for the charged fragments. The resulting gas‐phase EDA and solution‐phase SP EDA components are collected in Table 2.

**TABLE 2:** Vacuum EDA and SP‐EDA energy terms (in kcal/mol) for the Na^+^–OH^−^ complex with binding energies and Na˙˙˙O bond distance (in Å).^a^

SP‐EDA (EDA)	Na^+^˙˙˙OH^—^			
	COSMO			Vac.
	S1	S2	S3	V1
Δ*V*_elstat,S4‐SX_ (in vac. Δ*V*_elstat_)	−186.6	−184.1	−181.4	−178.8
Δ*E*_Pauli,S4‐SX_ (in vac. Δ*E*_Pauli_)	36.2	35.8	35.1	31.8
Δ*E*_oi,S4‐SX_ (in vac. Δ*E*_oi_)	−11.2	−12.7	−12.9	−18.6
Δ*E* _disp_	−1.1	−1.1	−1.1	−0.8
Δ*E*_DFT,S4‐SX_ (or Δ*E*_int,V4‐V1_)^b^	−162.7	−162.1	−160.3	−166.5
Δ*E* _solv,S4‐SX_	141.5	124.5	−93.8	—
Δ*E*_int,S4‐SX_ (or Δ*E*_int,V4‐V1_)^c^	−21.2	−37.6	−254.1	−166.5
Δ*E*_DESOLV, SX‐S1_	0.0	16.5	233.0	—
Δ*E* _strain,M1‐M0_ (M = S or V) ^d^	28.7			0.0
Δ*E* ^e^	7.6			−166.5
*Na*˙˙˙*O dist*.	2.02			2.02

^a^ Computed at BLYP‐D/TZ2P. See Figure 2 for a graphical illustration of all energy terms.

^b^ Sum of the four EDA terms above this entry: *c.f*. (Equation 10d) (and Equation 2).

^c^ Sum of the two terms above this entry: *c.f*. Equation (10a), (10b), (10c) (and Equation 7d).

^d^ In the solution‐phase analysis, the strain energy comes nearly entirely from the change in cavitation as the sodium radius increases, going from Na^+^ to NaOH.

^e^ Bond energy (sum of the three terms above this entry: *c.f*. Equation 7a): the difference between states S4 and S0 (or V4 and V0 for vacuum).

We begin with the overall bond energy, ΔE. In the gas phase, ΔE is strongly negative, as expected for the formation of an ion pair stabilized by the large Coulomb attraction between Na^+^ and OH^—^. In aqueous solution, by contrast, ΔE becomes positive. This is the physically correct result: it reflects the experimentally observed tendency of NaOH to dissociate spontaneously into Na^+^ and OH^—^ in solution. Thus, already at the level of the total bond energy, the solvent model changes the qualitative thermodynamic picture from ion‐pair formation in the gas phase to ion separation in solution.

The strain energy deserves a short comment because its meaning differs between the gas‐phase and SP EDA calculations. In the gas phase, the strain energy is essentially zero. This follows from the fact that the O–H bond length in OH^−^ changes by less than 0.01 Å between the fully optimized isolated anion and the distorted fragment geometry used in the EDA, corresponding to an energy change of less than 0.05 kcal mol^−1^, which rounds to Δ*E*_strain,M1‐M0_ = 0.0 in Table 2. In the SP‐EDA analyses, however, the strain term also includes the energetic effect associated with changes in the COSMO solvent cavity of sodium. Specifically, the cavity radius changes from the value for isolated Na^+^ in state S0 (1.62 Å) to the default Allinger radius of 2.25 Å used for states S1—S4. This radius adaptation is not just a technical detail but a necessary element of a physically meaningful phenomenon that needs to be accounted for also in a continuum‐solvation treatment: the effective size of an atom depends strongly on its charge state. A more extensive discussion of this point can be found in Ref. [28] and references cited therein.

Turning to the results obtained with the different solvation states (S1, S2, and S3), we find that the changes in the electrostatic, Pauli, and orbital interaction components [the components of Δ*E*_DFT,S4‐SX_ (X = 1, 2, or 3)] are of the same order of magnitude as in the charged water dimers. For example, in the H_3_O^+^•••H_2_O system, the differences between the S1 and S3 results for the electrostatic, Pauli, and orbital interaction terms are 8.6, −1.5, and −6.4 kcal/mol, respectively, whereas in the present oppositely charged system, they are 5.2, −1.1, and −1.7 kcal/mol, respectively. At the same time, these changes are about one order of magnitude smaller in the system with neutral fragments (H_2_O•••H_2_O), so the presence of two opposite charges does not qualitatively change the trends of the aforementioned EDA terms with respect to solvation state compared to the charged water dimers. A notable change is observed only in the absolute values of the Δ*V*_elstat,S4‐SX_ term in comparison to the charged water dimers. This is a direct consequence of the very strong Coulomb interaction between two ions in Na^+^•••OH^−^ compared to the interaction between an ion and a neutral in the charged water complexes. Overall, only relatively small changes across the different solvation states are observed in the electrostatic, Pauli, and orbital interaction terms (the dispersion correction only depends on atom types and geometry and therefore remains essentially constant). The reason is that the mutual exposure of the fragments to the potentials of the net +1 and −1 charged fragments is so strong that the effects of the different mirror‐charge distributions in states S1—S3 (although certainly present) are relatively small.

Finally, concerning the remaining terms, we can now draw a clear picture of the role of solvation effects. The Δ*E*_DFT,S4‐SX_ (X = 1, 2, 3) values from SP‐EDA are close to the corresponding Δ*E*_int,V4‐V1_ value in vacuum and indicate a strong interaction mainly originating from the electrostatic contribution. When using the S1 or S2 states, Δ*E*_DFT,S4‐SX_ (*X* = 1, 2) is largely compensated by the solvent term (Δ*E*_solv,S4‐SX_, *X* = 1,2,3) which represents the work required to rearrange the COSMO surface charges (and, for S1, the cavity enlargement, as well) from their fragment values to those appropriate for the complex. This is indeed a considerable contribution, because for both fragments the system must work against the strong electrostatic attraction between the induced COSMO charges and the charged fragment. A minor but noteworthy point is that the energy change associated with the change in solvent radius for the cation (included in the strain energy) is also a non‐negligible contribution and is required to obtain experimentally correct results, such as a positive ∆*E* at an Na—O distance corresponding to the equilibrium distance in the gas phase (i.e., the system is unbound in aqueous solution).

In contrast, when using the S3 state, we encounter exactly the same situation as in the water dimer cases: the extremely large positive Δ*E*_DESOLV,S3‐S1_ value reflects the unphysical nature of a state in which a solute fragment interacts with a solvent that has already adopted its configuration to the complex. In other words, the fragment experiences the mirror charges and cavity corresponding to the solute complex rather than to the isolated fragment. This again leads us to conclude that the S1 and S2 states are appropriate for obtaining a physically well‐defined and interpretable solution‐phase EDA, whereas S3 should be avoided.

## 5 Application 3: Guanine Tetramer

**FIGURE 4:**
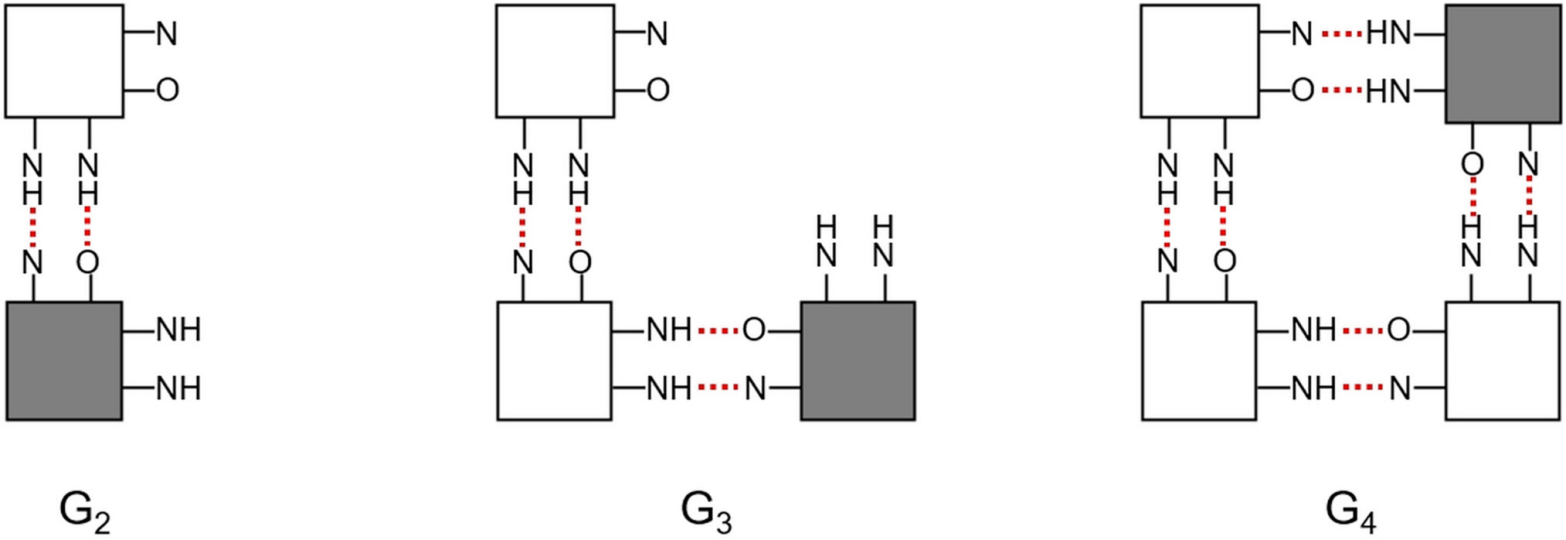
Stepwise assembly of the guanine tetramer G_4_.

In our third test set, we examine how the SP‐EDA analysis of the bonding in the guanine tetramer (G_4_) depends on choosing either the S1 or S2 solvation states. The G_4_ system features interesting and challenging phenomena, which we use to test our approach: G_4_ shows strong cooperativity effects in the gas phase, that is, the effective interaction between G monomers becomes stronger along G_2_, G_3_, and G_4_ (see Figure 4 and Table 3). In aqueous solution, the overall stability of G_4_ relative to its monomers is reduced and, most interestingly, the aforementioned cooperativity effects are significantly diminished [22]. First, we focus on these main trends before diving into the differences and similarities of the SP‐EDA energy components if one uses either the S1 or S2 solution states.

**TABLE 3:** Vacuum EDA and SP‐EDA energy terms^a^ (in kcal/mol) in the step‐by‐step construction of the guanine tetramer.

SP‐EDA (EDA)	G + G			G_2_ + G			G_3_ + G		
	COSMO		Vac.	COSMO		Vac.	COSMO		Vac.
	S1	S2	V1	S1	S2	V1	S1	S2	V1
Δ*V*_elstat,S4‐SX_ (in vac. Δ*V*_elstat_)	−36.2	−32.8	−25.9	−40.5	−36.8	−30.4	−78.5	−70.7	−59.9
Δ*E*_Pauli,S4‐SX_ (in vac. Δ*E*_Pauli_)	30.3	30.1	30.0	30.7	30.5	30.5	60.7	60.3	59.5
Δ*E*_oi,S4‐SX_ (in vac. Δ*E*_oi_)	−10.5	−10.5	−16.0	−12.8	−13.0	−18.2	−29.7	−31.3	−41.0
Δ*E* _disp_	−4.3	−4.3	−4.2	−4.5	−4.5	−4.3	−8.7	−8.7	−8.5
Δ*E*_DFT,S4‐SX_ (or Δ*E*_int,V4‐V1_) ^b^	−20.7	−17.5	−16.0	−27.0	−23.7	−22.4	−56.2	−50.4	−49.9
Δ*E* _solv,S4‐SX_	12.0	1.3	0.0	18.4	6.1	0.0	38.1	17.0	0.0
Δ*E*_int,S4‐SX_ (or Δ*E*_int,V4‐V1_) ^c^	−8.7	−16.2	−16.0	−8.6	−17.6	−22.4	−18.1	−33.4	−49.9
Δ*E*_DESOLV,SX‐S1_	0.0	7.5	—	0.0	8.9	—	0.0	15.3	—

^a^ Computed at BLYP‐D/TZ2P. See Figure 2 for a graphical illustration of all energy terms.

^b^ Sum of the four EDA terms above this entry: *c.f*. Equation 10d (and Equation 2).

^c^ Sum of the two terms above this entry: *c.f*. Equation (10a), (10b), (10c) (and Equation 7d).

In the gas phase, the interaction ∆*E*_int_ upon adding a guanine base stabilizes from G + G to G_2_ + G to G_3_ + G from −16.0 to −22.4 to −49.9 kcal/mol (see Table 3). The reason is an increasing charge separation across the G_n_ fragment as the latter becomes larger, due to HOMO–LUMO interactions in the hydrogen bonds between G monomers in G_n_ [22]. This effect enhances both the electrostatic and the HOMO–LUMO orbital interactions with the following G monomer that is added, hence the strong cooperativity effects in the gas phase. Upon solvation in water, this changes.

The main effect of solvation, whether one takes S1 or S2 reference states, is a weakening of the bond energy ∆*E* between G monomers. This weakening in ∆*E* stems mainly from the destabilizing differential solvation term Δ*E*_solv,S4‐SX_, not from the interaction energy Δ*E*_DFT,S4‐SX_ (see Table 3). Interestingly, the latter is even somewhat more stabilizing in solution than in the gas phase. The loss of cooperativity effects in aqueous solution is also caused by the differential solvation term Δ*E*_solv,S4‐SX_ not by the intrinsic interaction Δ*E*_DFT,S4‐SX_. The latter still becomes more stabilizing along G_2_, G_3_, and G_4_. However, the trend of increasing stabilization in Δ*E*_DFT,S4‐SX_ is counteracted by a similar aggravation in the destabilizing differential solvation Δ*E*_solv,S4‐SX_.

Taking the S1 state, for example, ∆*E*_DFT,S4‐S1_ goes from −20.7 kcal/mol for G + G in G_2_ to −27.0 kcal/mol for G_2_ + G in G_3_, a stabilization that is compensated by the destabilization in ∆*E*_solv,S4‐S1_ which goes from 12.0 to 18.4 kcal/mol, respectively. In the following, we examine the origins of these trends in the various interaction components.

**FIGURE 5:**
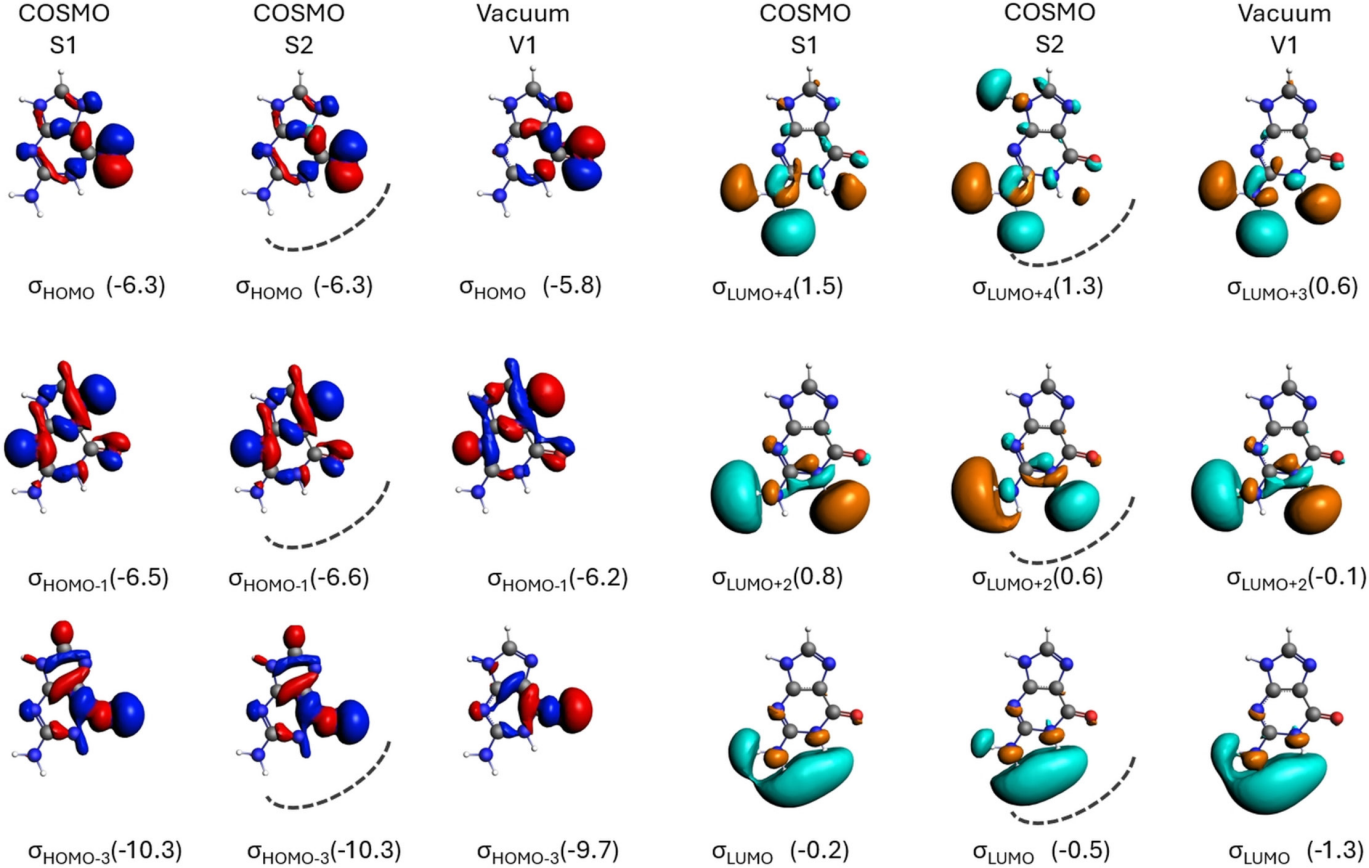
Guanine (G) MOs and MO energies (in eV) in S1, S2, and vacuum (V1) states, computed at (COSMO‐)BLYP‐D/TZ2P (G is in the structure it adopts in G_2_ in aqueous solution or in vacuum). For the solvation S2 state, the dashed lines indicate the site at which the cavity is enlarged (see Figure S1 for quantitative representations of COSMO surfaces for σ_HOMO_ and σ_LUMO_).

**FIGURE 6:**
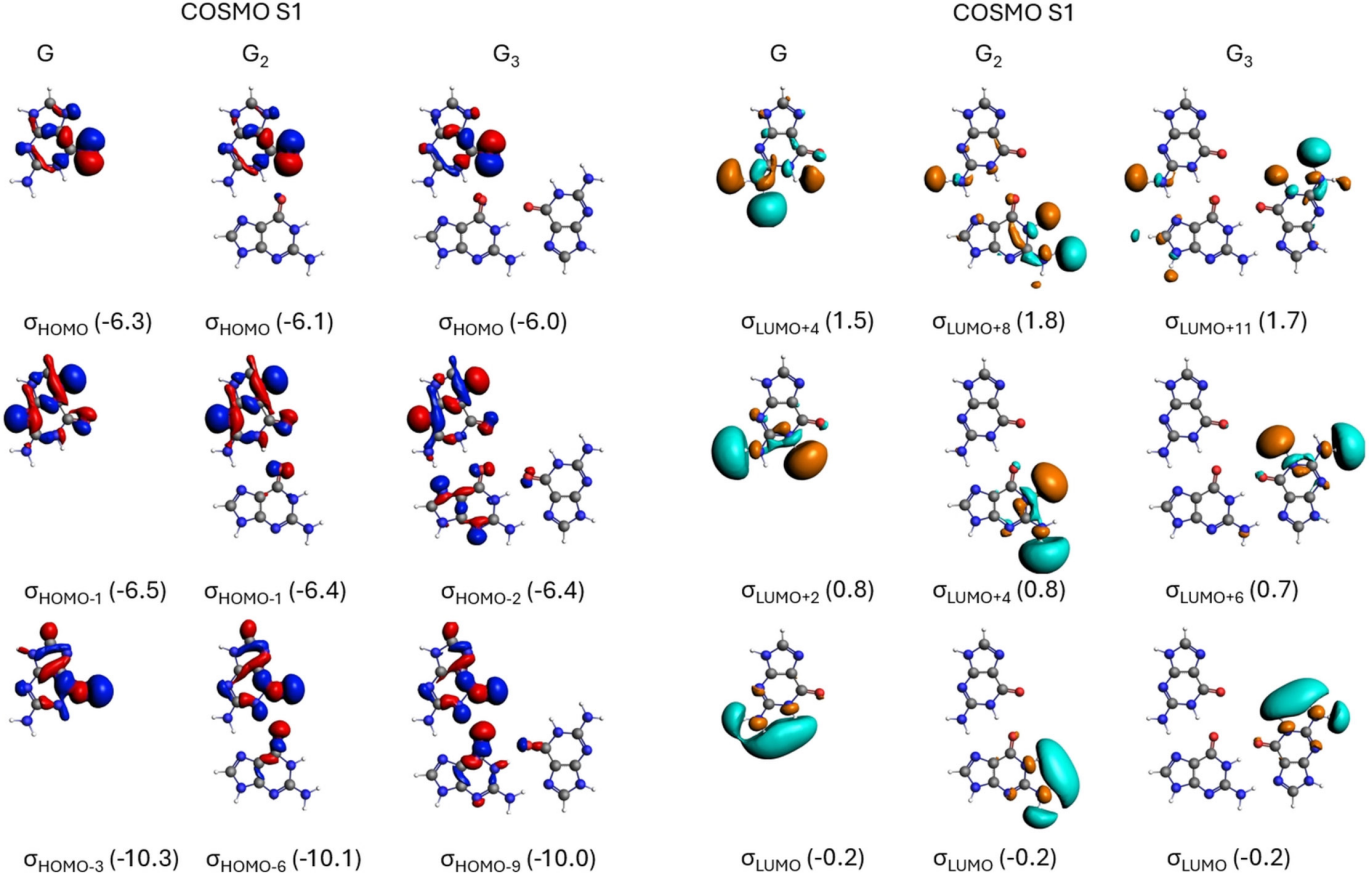
G (guanine), G_2_, and G_3_ MOs and MO energies (in eV) in the solvation S1 state of the SP‐EDA method, computed at COSMO‐BLYP‐D/TZ2P (all systems are in the structure they adopt in G_4_ in aqueous solution).

**FIGURE 7:**
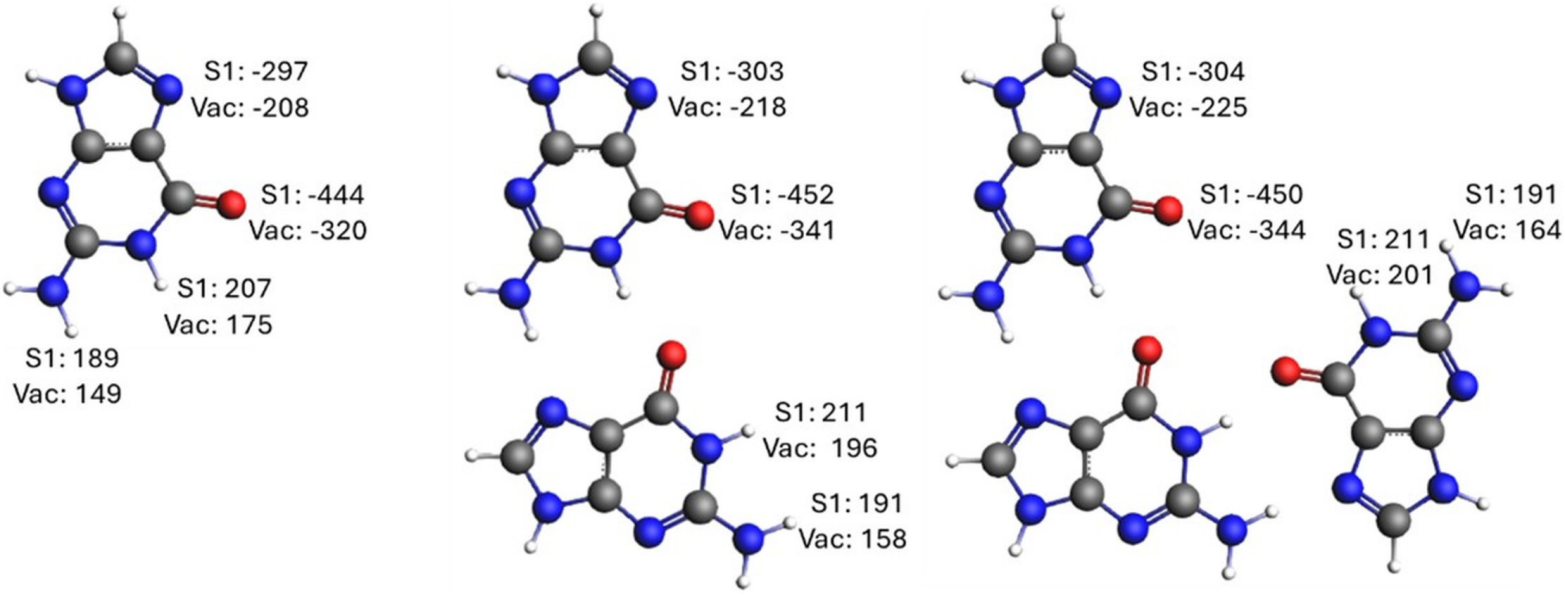
VDD atomic charges (in milli a.u.) of selected atoms in G, G_2_, and G_3_, computed in the S1 solvation state and in vacuum at (COSMO‐)BLYP‐D/TZ2P (all systems are in the structure they adopt in G_4_ in aqueous solution or in vacuum, respectively).

Solvation stabilizes the occupied orbitals of guanine and its complexes, as shown in Figures 5 and 6. For example, the σ_HOMO_ of the G monomer drops in energy from −5.8 eV in vacuum to −6.3 eV in the S1 and S2 solvation states. In the course of the solute–solvent interaction, the solvent medium (or, more precisely, the mirror charges on the cavity surface) is configured for optimal solvation interaction with the charge density associated with the occupied orbitals. However, the medium is therefore not optimally arranged to stabilize the unoccupied orbitals, which are, as a result, somewhat destabilized. For example, the σ_LUMO_ orbital energy of the G monomer rises from −1.3 eV in vacuum to −0.2 and −0.5 eV for the S1 and S2 solvation states, respectively. Overall, the result is a widening of the HOMO–LUMO gap and thus a weakening of the orbital interactions Δ*E*_oi,S4‐SX_ in aqueous solution. Differences in the Δ*E*_oi,S4‐SX_ terms if one uses solvation states S1 or S2 are minor, for example, 0.0 kcal/mol for G + G, 0.2 kcal/mol for G_2_ + G, and 1.6 kcal/mol for G_3_ + G (see Table 3). Furthermore, solvation stabilizes charge separation and thus slightly enhances polarization within the guanine monomer, as shown in Figure 7. For example, guanine's hydrogen‐bond accepting nitrogen atom exhibits VDD atomic charges of −208, −218, and − 225 milli a.u. in vacuum and becomes more negative in the S1 solvation state with VDD atomic charges of −297, −303, and −304 milli a.u. (see Figure 7). The enhanced polarization within the guanine monomer, in turn, stabilizes the G–G electrostatic attraction Δ*V*_elstat,S4‐SX_ (see Table 3). The effect is slightly more pronounced for the solvation state S1 than for state S2, but the differences do not lead to qualitatively different trends or conclusions. Although the change in the shape of the slightly polarized occupied orbitals in solution is small, the concomitant stabilization of Δ*V*_elstat,S4‐SX_ outweighs the weakening in Δ*E*_oi,S4‐SX_. The alteration in the shape of the occupied orbitals is indeed minor in that it can be barely perceived with the naked eye (see Figures 5 and 6). In line with this, there is essentially no effect on the Pauli repulsion between the occupied orbitals of G_n_ and those of G, which in all situations amounts to ca 30 kcal/mol per G–G contact. Differences between solvation states S1 and S2 are minor.

Solvation also introduces the aforementioned differential solvation term Δ*E*_solv,S4‐SX_. This term is destabilizing and weakens the overall complexation energy ∆*E* because of the partial desolvation upon complexation of the solute monomers. This desolvation becomes energetically more unfavorable in the larger G_n_ fragments because they feature a larger charge separation due to the internal hydrogen bonds that have already been formed. This trend counteracts the enhanced intrinsic interaction occurring for larger G_n_ fragments, which dampens the cooperativity effects.

In conclusion, solvation weakens the interaction in guanine aggregates mainly by weakening orbital interactions, and it damps cooperativity mainly by differential solvation effects. The dependence of SP‐EDA terms on the solvation state S1 (cavity of fragment) versus solvation state S2 is minor, and both states can therefore be used for obtaining physically reasonable SP‐EDA terms. An advantage of state S1 is its robustness in computational applications because of the straightforward definition of fragment cavities.

## 6 Conclusion

We have extended the canonical energy decomposition analyses (EDA) of the interaction between molecular fragments from bonding in the gas phase (EDA) to bonding in the solution phase (SP‐EDA) using the Conductor‐like Screening Model (COSMO) to simulate the effect of the solvent, with applications to simple neutral and ionic complexes involving water, sodium‐hydroxide, as well as guanine aggregates.

In the SP‐EDA approach, the interaction between fragments in solution $\Delta E_{\mathrm{int},S4-\mathrm{SX}}$ consists of the actual solute–solute interaction $\Delta E_{\mathrm{DFT},S4-\mathrm{SX}}$ plus the differential solvation $\Delta E_{\text{solv},S4-\mathrm{SX}}$, that is, the difference in solvation of the solute complex and the solvation of the solute fragments: $\Delta E_{\mathrm{int},S4-\mathrm{SX}}=\Delta E_{\mathrm{DFT},S4-\mathrm{SX}}+\Delta E_{\text{solv},S4-\mathrm{SX}}$ (see Equations 10). Here, the states SX play a key role: S1 refers to the separate reactants in the geometry they adopt in the complex and associated modified solvent cavity, S2 to the same fragments in the cavity of the overall complex (this can be interpreted as the partial desolvation needed to form the complex), and S4 to the overall complex in its associated cavity. We have also defined a state S3, which behaves erratically and is physically problematic (see discussion).

The SP‐EDA method has been successfully applied to the model systems mentioned above. It shows, for example, how solvation weakens orbital interactions in the ionic water complexes and in the guanine dimer, trimer, and tetramer, and how differential solvation, not orbital interactions, dampens the cooperativity in the guanine tetramer formation. A more general conclusion for future applications is that the dependence of SP‐EDA terms ($\Delta V_{\text{elstat},S4-\mathrm{SX}},\Delta E_{\text{Pauli},S4-\mathrm{SX}},\Delta E_{\mathrm{oi},S4-\mathrm{SX}}$) on the intermediate state S1 (cavity of fragment) versus intermediate state S2 (cavity of overall complex) is minor. Both states can therefore be used to obtain physically reasonable SP‐EDA terms. An advantage of state S1 is its robustness in computational applications because of the straightforward definition of fragment cavities.

## 7 Computational Details

All calculations were carried out with a development version of the Amsterdam Density Functional program, ADF2016 [29, 30, 31]. Equilibrium geometries in vacuum and implicit solvent were optimized using the BLYP functional augmented with the dispersion correction suggested by Grimme [32, 33, 34], that is, at the BLYP‐D level. In the case of tetramers, planar symmetry constraints were applied, and for the NaOH structure the Na—O distance was kept fixed at the vacuum‐optimized value during the COSMO optimization. In all cases, the all‐electron TZ2P basis set was used (i.e., no frozen‐core approximation). The TZ2P basis set consists of a large, uncontracted set of Slater‐type orbitals of triple‐ζ quality, augmented by two sets of polarization functions: d and f on heavy atoms, 2p and 3d sets on H [29, 30, 31]. The Becke grid integration [35] and ZLMfit method [36] were set to normal quality in the self‐consistent field (SCF) procedure. Vibrational analyses confirmed that all stationary points are equilibrium geometries. Cartesian coordinates and ADF total energies of all species involved in this study can be found in Tables S1–S4 in the Supporting Information. The original COSMO implementation [37] in the ADF code was adjusted so that the modified program can perform the SCF procedure for the S2 or S3 states. In COSMO, the solvent cavity was created according to the Delley method [38], applying the default Allinger parameters [39] for atomic radii provided by the ADF code. The radius of the Na^+^ cation at S0 state was modified to 1.62 Å, in line with our previous calculations [40], to provide a reliable solvation energy by reproducing the experimental value of −101.3 kcal/mol [41]. Finally, charge analyses were performed using the Voronoi Deformation Density (VDD) method, as implemented in the ADF program [42, 43].

## Funding

This work was supported by the National Research, Development and Innovation Fund, TKP2021‐EGA‐17; Nederlandse Organisatie voor Wetenschappelijk Onderzoek.

## Supporting information


**Figure S1:** Quantitative representations of the full COSMO surfaces with σ_HOMO_ (left) and σ_LUMO_ (right) orbitals at the S2 solvation state calculation for the guanine fragment in a dimer calculation, computed at COSMO‐BLYP‐D/TZ2P. Red, blue, and white indicate regions of positive, negative, and near‐neutral charge densities, respectively (see color scale in e/Å^2^ unit). In Figure 5 of the main text, we indicated the site at which the cavity is enlarged only schematically with a dashed line.
**Table S1:** Cartesian coordinates (in Å) and ADF total energies *E* (in kcal/mol) of water and sodium hydroxide complexes and the corresponding fragment molecules in vacuum, computed at BLYP‐D/TZ2P.
**Table S2:** Cartesian coordinates (in Å) and ADF total energies *E* (in kcal/mol) of water and sodium hydroxide complexes and the corresponding fragment molecules in aqueous solution, computed at COSMO‐BLYP‐D/TZ2P. Default Allinger parameters were used for the COSMO radii, except for the separated Na^+^ cation at the S0 state (see Computational Details).
**Table S3:** Cartesian coordinates (in Å) of the guanine tetramer in vacuum and in C_4h_ symmetry, computed at BLYP‐D/TZ2P. The coordinates of the monomer, the dimer complex, and the trimer complex in vacuum were taken from this structure. The last column shows the unit identifier belonging to a given atom in the guanine tetramer consisting of 4 units.
**Table S4:** Cartesian coordinates (in Å) of the guanine tetramer in aqueous solution and in C_4h_ symmetry, computed at COSMO‐BLYP‐D/TZ2P. The coordinates of the monomer, the dimer complex, and the trimer complex in vacuum were taken from this structure. The last column shows the unit identifier belonging to a given atom in the guanine tetramer consisting of 4 units.

## Data Availability

All data that is needed for reproducibility is contained in the manuscript (in particular, the Computational Details) and in the Supporting Information (among others, Cartesian coordinates and ADF total energies of molecular and ionic species).
