# Supplementary material for: Solution‐Phase Energy Decomposition Analysis (SP‐EDA): Theory and Applications
Source: J Comput Chem. 2026 Jul 17;47(20):e70451. doi: 10.1002/jcc.70451 (PMC13379323; doi:10.1002/jcc.70451)
Supplement: Supplementary file 1 — Figure S1: Quantitative representations of the full COSMO surfaces with σHOMO (left) and σLUMO (right) orbitals at the S2 solvation state calculation for the guanine fragment in a dimer calculation, computed at COSMO‐BLYP‐D/TZ2P. Red, blue, and white indicate regions of positive, negative, and near‐neutral charge densities, respectively (see color scale in e/Å2 unit). In Figure 5 of the main text, we indicated the site at which the cavity is enlarged only schematically with a dashed line. Table S1: Cartesian coordinates (in Å) and ADF total energies E (in kcal/mol) of water and sodium hydroxide complexes and the corresponding fragment molecules in vacuum, computed at BLYP‐D/TZ2P. Table S2: Cartesian coordinates (in Å) and ADF total energies E (in kcal/mol) of water and sodium hydroxide complexes and the corresponding fragment molecules in aqueous solution, computed at COSMO‐BLYP‐D/TZ2P. Default Allinger parameters were used for the COSMO radii, except for the separated Na+ cation at the S0 state (see Computational Details). Table S3: Cartesian coordinates (in Å) of the guanine tetramer in vacuum and in C4h symmetry, computed at BLYP‐D/TZ2P. The coordinates of the monomer, the dimer complex, and the trimer complex in vacuum were taken from this structure. The last column shows the unit identifier belonging to a given atom in the guanine tetramer consisting of 4 units. Table S4: Cartesian coordinates (in Å) of the guanine tetramer in aqueous solution and in C4h symmetry, computed at COSMO‐BLYP‐D/TZ2P. The coordinates of the monomer, the dimer complex, and the trimer complex in vacuum were taken from this structure. The last column shows the unit identifier belonging to a given atom in the guanine tetramer consisting of 4 units. [file JCC-47-0-s001.docx]

Supporting Information for

Solution-Phase Energy Decomposition Analysis (SP-EDA):

Theory and Applications

Gábor Paragi,* ^[a,b,c]^ Célia Fonseca Guerra,*^[d]^ and F. Matthias Bickelhaupt*^[d,e,f]^

[a] Dr. G. Paragi
Institute of Physics
University of Pécs
Ifjúság útja 6, Pécs, Hungary, H-7624

[b] Dr. G. Paragi
Department of Theoretical Physics
Tisza Lajos krt. 84-86, Szeged, Hungary, H-6720

[c] Dr. G. Paragi
Institute of Medicinal Chemistry
University of Szeged
Dóm tér 8, Szeged, Hungary, H-6720

[d] Prof. Dr. F. M. Bickelhaupt, Prof. Dr. C. Fonseca Guerra
Department of Chemistry and Pharmaceutical Sciences
Vrije Universiteit Amsterdam
De Boelelaan 1108, NL-1081 HZ Amsterdam, The Netherlands
E-mail: f.m.bickelhaupt@vu.nl, c.fonsecaguerra@vu.nl

[e] Prof. Dr. F. M. Bickelhaupt

Institute of Molecules and Materials
Radboud University
Heyendaalseweg 135, NL-6525 AJ Nijmegen, The Netherlands

[f] Prof. Dr. F. M. Bickelhaupt

Department of Chemical Sciences

University of Johannesburg

Auckland Park, Johannesburg 2006, South Africa

**Contents**

**Figure S1.** Quantitative representations of the full COSMO surfaces with σ_HOMO_ (left) and σ_LUMO_ (right) orbitals at the S2 solvation state calculation for the guanine fragment in a dimer calculation, computed at COSMO-BLYP-D/TZ2P. Red, blue, and white indicate regions of positive, negative, and near-neutral charge densities, respectively (see color scale in e/Å² unit). In Figure 5 of the main text, we indicated the site at which the cavity is enlarged only schematically with a dashed line.

**Table S1.** Cartesian coordinates (in Å) and ADF total energies *E* (in kcal/mol) of water and sodium hydroxide complexes and the corresponding fragment molecules in vacuum, computed at BLYP-D/TZ2P.

**Table S2.** Cartesian coordinates (in Å) and ADF total energies *E* (in kcal/mol) of water and sodium hydroxide complexes and the corresponding fragment molecules in aqueous solution, computed at COSMO-BLYP-D/TZ2P. Default Allinger parameters were used for the COSMO radii, except for the separated Na⁺ cation at the S0 state (see Computational Details).

**Table S3.** Cartesian coordinates (in Å) of the guanine tetramer in vacuum and in C_4h_ symmetry, computed at BLYP-D/TZ2P. The coordinates of the monomer, the dimer complex, and the trimer complex in vacuum were taken from this structure. The last column shows the unit identifier belonging to a given atom in the guanine tetramer consisting of four units.

**Table S4.** Cartesian coordinates (in Å) of the guanine tetramer in aqueous solution and in C_4h_ symmetry, computed at COSMO-BLYP-D/TZ2P. The coordinates of the monomer, the dimer complex, and the trimer complex in vacuum were taken from this structure. The last column shows the unit identifier belonging to a given atom in the guanine tetramer consisting of four units.

**
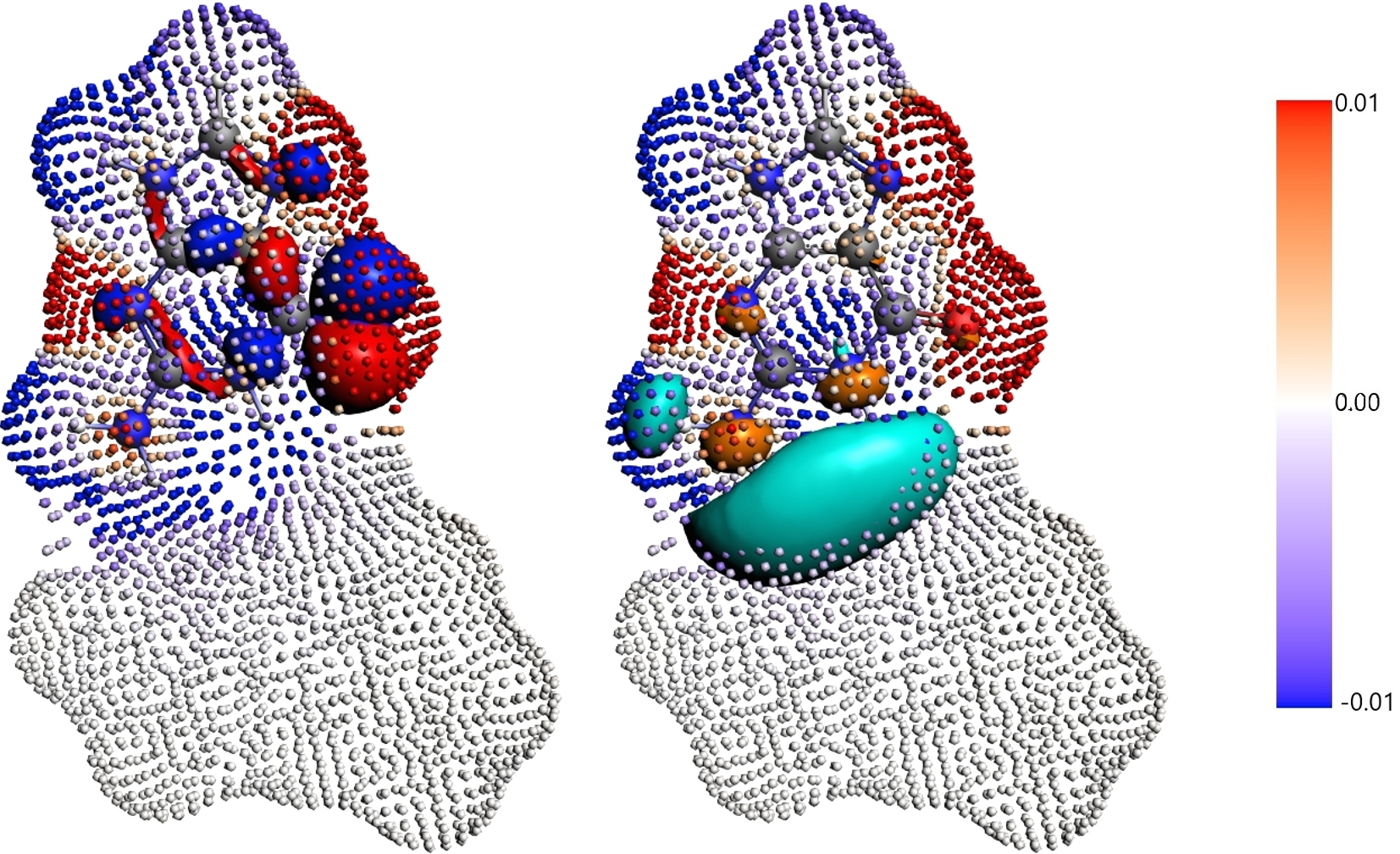
**

**Figure S1.** Quantitative representations of the full COSMO surfaces with σ_HOMO_ (left) and σ_LUMO_ (right) orbitals at the S2 solvation state calculation for the guanine fragment in a dimer calculation, computed at COSMO-BLYP-D/TZ2P. Red, blue, and white indicate regions of positive, negative, and near-neutral charge densities, respectively (see color scale in e/Å² unit). In Figure 5 of the main text, we indicated the site at which the cavity is enlarged only schematically with a dashed line.

**Table S1.** Cartesian coordinates (in Å) and ADF total energies *E* (in kcal/mol) of water and sodium hydroxide complexes and the corresponding fragment molecules in vacuum, computed at BLYP-D/TZ2P.

| H_2_O•••H_2_O | | *E* = | -640.70 |  | Na^+^•••HO ^–^ | | *E* = | -252.47 |
| --- | --- | --- | --- | --- | --- | --- | --- | --- |
| O | -1.465 | -0.095 | -0.039 |  | Na | 0.000 | 0.000 | 0.000 |
| H | -1.659 | 0.082 | 0.899 |  | O | 2.016 | 0.000 | 0.000 |
| H | -1.678 | 0.737 | -0.498 |  | H | 2.616 | -0.760 | 0.000 |
| O | 1.417 | 0.071 | 0.071 |  |  | |  |  |
| H | 1.847 | -0.711 | -0.316 |  | HO ^–^ |  | *E* = | -206.32 |
| H | 0.455 | -0.096 | -0.028 |  | O | 0.000 | 0.000 | 1.481 |
|  |  |  |  |  | H | 0.000 | 0.000 | 0.503 |
| H_3_O^+^•••H_2_O | | *E* = | -550.43 |  |  |  |  |  |
| O | -1.626 | -0.174 | 0.202 |  |  | |  |  |
| H | -1.996 | 0.344 | 0.947 |  | H_2_O |  | *E* = | -317.64 |
| H | -2.198 | -0.072 | -0.586 |  | O | 0.000 | 0.000 | 0.794 |
| O | 0.798 | -0.195 | 0.029 |  | H | -0.769 | 0.000 | 0.198 |
| H | 1.276 | 0.392 | -0.593 |  | H | 0.769 | 0.000 | 0.198 |
| H | 1.220 | -1.080 | 0.034 |  |  |  |  |  |
| H | -0.417 | -0.157 | 0.058 |  |  |  |  |  |
|  |  |  |  |  | H_3_O^+^ |  | *E* = | -195.72 |
| HO ^–^ •••H_2_O | | *E* = | -559.28 |  | O | 0.000 | 0.000 | 0.293 |
| O | -1.233 | -0.162 | -0.386 |  | H | 0.476 | 0.825 | 0.021 |
| H | -1.515 | 0.505 | 0.264 |  | H | 0.476 | -0.825 | 0.021 |
| H | 1.515 | -0.505 | 0.264 |  |  |  |  |  |
| O | 1.233 | 0.162 | -0.386 |  | Na^+^ |  | E= | 120.32 |
| H | 0.000 | 0.000 | -0.400 |  | Na | 0.000 | 0.000 | 0.000 |
|  |  |  |  |  |  |  |  |  |

**Table S2.** Cartesian coordinates (in Å) and ADF total energies *E* (in kcal/mol) of water and sodium hydroxide complexes and the corresponding fragment molecules in aqueous solution, computed at COSMO-BLYP-D/TZ2P. Default Allinger parameters were used for the COSMO radii, except for the separated Na⁺ cation at the S0 state (see Computational Details).

| H_2_O•••H_2_O | | *E* = | -651.06 |  | Na^+^•••HO ^–^ | | E = | | -277.84 |
| --- | --- | --- | --- | --- | --- | --- | --- | --- | --- |
| O | -1.371 | -0.084 | -0.025 |  | Na | 0.000 | 0.000 | | 0.000 |
| H | -1.685 | 0.066 | 0.886 |  | O | 2.016 | 0.000 | | 0.000 |
| H | -1.680 | 0.698 | -0.520 |  | H | 2.401 | -0.891 | | 0.000 |
| O | 1.438 | 0.061 | 0.082 |  |  |  |  | |  |
| H | 1.765 | -0.743 | -0.361 |  | HO ^–^ |  | *E* = | | -304.37 |
| H | 0.450 | -0.010 | 0.028 |  | O | 0.000 | 0.000 | | -0.460 |
|  |  |  |  |  | H | 0.000 | 0.000 | | -1.431 |
| H_3_O^+^•••H_2_O | | *E* = | -623.14 |  |  |  |  | |  |
| O | -1.626 | -0.245 | 0.222 |  | H_2_O | |  | *E* = | -323.53 |
| H | -1.963 | 0.369 | 0.906 |  | O | 0.000 | 0.000 | | 0.765 |
| H | 1.184 | -1.064 | -0.004 |  | H | 0.769 | 0.000 | | 1.366 |
| H | -0.412 | -0.191 | 0.123 |  | H | -0.769 | 0.000 | | 1.366 |
| O | 0.803 | -0.168 | 0.097 |  |  |  |  | |  |
| H | 1.151 | 0.380 | -0.637 |  | H_3_O^+^ |  | *E* = | | -283.78 |
| H | -2.082 | -0.023 | -0.616 |  | O | 0.000 | 0.000 | | -0.397 |
|  |  |  |  |  | H | -0.932 | 0.000 | | -0.077 |
| HO ^–^ •••H_2_O | | *E* = | -638.74 |  | H | -0.932 | 0.000 | | -0.077 |
| O | -1.894 | 0.203 | -0.016 |  | H | 0.466 | 0.807 | | -0.077 |
| H | -2.119 | 0.043 | 0.917 |  |  |  |  | |  |
| H | 0.994 | -0.372 | -0.255 |  | Na^+^ |  | E= | | 19.00 |
| H | -0.464 | 0.351 | -0.056 |  | Na | 0.000 | 0.000 | | 0.000 |
| O | 0.611 | 0.514 | -0.125 |  |  |  |  | |  |
|  |  |  |  |  |  |  |  | |  |

**Table S3.** Cartesian coordinates (in Å) of the guanine tetramer in vacuum and in C_4h_ symmetry, computed at BLYP-D/TZ2P. The coordinates of the monomer, the dimer complex, and the trimer complex in vacuum were taken from this structure. The last column shows the unit identifier belonging to a given atom in the guanine tetramer consisting of four units.

| N | 1.179 | 6.829 | 0.000 | gu1 |
| --- | --- | --- | --- | --- |
| O | 0.970 | 2.217 | 0.000 | gu1 |
| C | 2.455 | 6.278 | 0.000 | gu1 |
| N | 2.427 | 4.960 | 0.000 | gu1 |
| N | -0.977 | 3.467 | 0.000 | gu1 |
| C | -1.665 | 4.668 | 0.000 | gu1 |
| C | 1.078 | 4.625 | 0.000 | gu1 |
| N | -3.017 | 4.618 | 0.000 | gu1 |
| C | 0.279 | 5.777 | 0.000 | gu1 |
| H | -3.492 | 5.512 | 0.000 | gu1 |
| N | -1.065 | 5.868 | 0.000 | gu1 |
| H | 3.345 | 6.897 | 0.000 | gu1 |
| H | -3.586 | 3.756 | 0.000 | gu1 |
| H | 0.934 | 7.814 | 0.000 | gu1 |
| C | 0.440 | 3.339 | 0.000 | gu1 |
| H | -1.508 | 2.567 | 0.000 | gu1 |
| N | 6.829 | -1.179 | 0.000 | gu2 |
| O | 2.217 | -0.970 | 0.000 | gu2 |
| C | 6.278 | -2.455 | 0.000 | gu2 |
| N | 3.467 | 0.977 | 0.000 | gu2 |
| C | 4.668 | 1.665 | 0.000 | gu2 |
| C | 4.625 | -1.078 | 0.000 | gu2 |
| N | 4.618 | 3.017 | 0.000 | gu2 |
| N | 4.960 | -2.427 | 0.000 | gu2 |
| H | 7.814 | -0.934 | 0.000 | gu2 |
| H | 5.512 | 3.492 | 0.000 | gu2 |
| N | 5.868 | 1.065 | 0.000 | gu2 |
| H | 6.897 | -3.345 | 0.000 | gu2 |
| H | 3.756 | 3.586 | 0.000 | gu2 |
| C | 5.777 | -0.279 | 0.000 | gu2 |
| C | 3.339 | -0.440 | 0.000 | gu2 |
| H | 2.567 | 1.508 | 0.000 | gu2 |
| N | -1.179 | -6.829 | 0.000 | gu3 |
| O | -0.970 | -2.217 | 0.000 | gu3 |
| C | -2.455 | -6.278 | 0.000 | gu3 |
| C | -0.279 | -5.777 | 0.000 | gu3 |
| N | 0.977 | -3.467 | 0.000 | gu3 |
| C | 1.665 | -4.668 | 0.000 | gu3 |
| C | -1.078 | -4.625 | 0.000 | gu3 |
| N | 3.017 | -4.618 | 0.000 | gu3 |
| H | 3.492 | -5.512 | 0.000 | gu3 |
| N | 1.065 | -5.868 | 0.000 | gu3 |
| H | -3.345 | -6.897 | 0.000 | gu3 |
| H | 3.586 | -3.756 | 0.000 | gu3 |
| N | -2.427 | -4.960 | 0.000 | gu3 |
| H | -0.934 | -7.814 | 0.000 | gu3 |
| C | -0.440 | -3.339 | 0.000 | gu3 |
| H | 1.508 | -2.567 | 0.000 | gu3 |
| N | -6.829 | 1.179 | 0.000 | gu4 |
| O | -2.217 | 0.970 | 0.000 | gu4 |
| C | -6.278 | 2.455 | 0.000 | gu4 |
| N | -4.960 | 2.427 | 0.000 | gu4 |
| H | -7.814 | 0.934 | 0.000 | gu4 |
| N | -3.467 | -0.977 | 0.000 | gu4 |
| C | -4.668 | -1.665 | 0.000 | gu4 |
| C | -4.625 | 1.078 | 0.000 | gu4 |
| N | -4.618 | -3.017 | 0.000 | gu4 |
| C | -5.777 | 0.279 | 0.000 | gu4 |
| H | -5.512 | -3.492 | 0.000 | gu4 |
| N | -5.868 | -1.065 | 0.000 | gu4 |
| H | -6.897 | 3.345 | 0.000 | gu4 |
| H | -3.756 | -3.586 | 0.000 | gu4 |
| C | -3.339 | 0.440 | 0.000 | gu4 |
| H | -2.567 | -1.508 | 0.000 | gu4 |

**Table S4.** Cartesian coordinates (in Å) of the guanine tetramer in aqueous solution and in C_4h_ symmetry, computed at COSMO-BLYP-D/TZ2P. The coordinates of the monomer, the dimer complex, and the trimer complex in vacuum were taken from this structure. The last column shows the unit identifier belonging to a given atom in the guanine tetramer consisting of four units.

| N | 1.200 | 6.764 | 0.000 | gu1 |
| --- | --- | --- | --- | --- |
| O | 0.853 | 2.159 | 0.000 | gu1 |
| C | 2.453 | 6.179 | 0.000 | gu1 |
| N | 2.390 | 4.858 | 0.000 | gu1 |
| N | -1.054 | 3.471 | 0.000 | gu1 |
| C | -1.715 | 4.691 | 0.000 | gu1 |
| C | 1.029 | 4.564 | 0.000 | gu1 |
| N | -3.063 | 4.667 | 0.000 | gu1 |
| C | 0.271 | 5.746 | 0.000 | gu1 |
| H | -3.541 | 5.561 | 0.000 | gu1 |
| N | -1.075 | 5.871 | 0.000 | gu1 |
| H | 3.359 | 6.772 | 0.000 | gu1 |
| H | -3.630 | 3.798 | 0.000 | gu1 |
| H | 1.002 | 7.760 | 0.000 | gu1 |
| C | 0.350 | 3.310 | 0.000 | gu1 |
| H | -1.602 | 2.586 | 0.000 | gu1 |
| N | 6.764 | -1.200 | 0.000 | gu2 |
| O | 2.159 | -0.853 | 0.000 | gu2 |
| C | 6.179 | -2.453 | 0.000 | gu2 |
| N | 3.471 | 1.054 | 0.000 | gu2 |
| C | 4.691 | 1.715 | 0.000 | gu2 |
| C | 4.564 | -1.029 | 0.000 | gu2 |
| N | 4.667 | 3.063 | 0.000 | gu2 |
| N | 4.858 | -2.390 | 0.000 | gu2 |
| H | 7.760 | -1.002 | 0.000 | gu2 |
| H | 5.561 | 3.541 | 0.000 | gu2 |
| N | 5.871 | 1.075 | 0.000 | gu2 |
| H | 6.772 | -3.359 | 0.000 | gu2 |
| H | 3.798 | 3.630 | 0.000 | gu2 |
| C | 5.746 | -0.271 | 0.000 | gu2 |
| C | 3.310 | -0.350 | 0.000 | gu2 |
| H | 2.586 | 1.602 | 0.000 | gu2 |
| N | -1.200 | -6.764 | 0.000 | gu3 |
| O | -0.853 | -2.159 | 0.000 | gu3 |
| C | -2.453 | -6.179 | 0.000 | gu3 |
| C | -0.271 | -5.746 | 0.000 | gu3 |
| N | 1.054 | -3.471 | 0.000 | gu3 |
| C | 1.715 | -4.691 | 0.000 | gu3 |
| C | -1.029 | -4.564 | 0.000 | gu3 |
| N | 3.063 | -4.667 | 0.000 | gu3 |
| H | 3.541 | -5.561 | 0.000 | gu3 |
| N | 1.075 | -5.871 | 0.000 | gu3 |
| H | -3.359 | -6.772 | 0.000 | gu3 |
| H | 3.630 | -3.798 | 0.000 | gu3 |
| N | -2.390 | -4.858 | 0.000 | gu3 |
| H | -1.002 | -7.760 | 0.000 | gu3 |
| C | -0.350 | -3.310 | 0.000 | gu3 |
| H | 1.602 | -2.586 | 0.000 | gu3 |
| N | -6.764 | 1.200 | 0.000 | gu4 |
| O | -2.159 | 0.853 | 0.000 | gu4 |
| C | -6.179 | 2.453 | 0.000 | gu4 |
| N | -4.858 | 2.390 | 0.000 | gu4 |
| H | -7.760 | 1.002 | 0.000 | gu4 |
| N | -3.471 | -1.054 | 0.000 | gu4 |
| C | -4.691 | -1.715 | 0.000 | gu4 |
| C | -4.564 | 1.029 | 0.000 | gu4 |
| N | -4.667 | -3.063 | 0.000 | gu4 |
| C | -5.746 | 0.271 | 0.000 | gu4 |
| H | -5.561 | -3.541 | 0.000 | gu4 |
| N | -5.871 | -1.075 | 0.000 | gu4 |
| H | -6.772 | 3.359 | 0.000 | gu4 |
| H | -3.798 | -3.630 | 0.000 | gu4 |
| C | -3.310 | 0.350 | 0.000 | gu4 |
| H | -2.586 | -1.602 | 0.000 | gu4 |
